# A Spectral-Emissivity-Corrected Method for Temperature Inversion from CCD Images

**DOI:** 10.3390/s26175461

**Published:** 2026-08-28

**Authors:** Meng Zhao, Chunyu Liu, Maoyong Bai, Zheng Qiu, Shaodong Bai, Kang Du, Yong Tan, Hongxing Cai

**Affiliations:** Key Laboratory of Spectral Detection Science and Technology, School of Physics, Changchun University of Science and Technology, Changchun 130022, China

**Keywords:** high-temperature field measurement, spectral-image fusion, integration-time-dependent radiometric calibration, spectral emissivity transfer, multiwavelength thermometry, genetic algorithm, pixelwise temperature inversion

## Abstract

Accurate high-temperature field characterization is important for explosion diagnostics, laser–matter interaction, combustion monitoring, and related thermal processes. This work presents an integrated thermometry framework combining fiber-optic spectrometry with monochrome imaging. Its central contribution is not a new multispectral principle or optimization algorithm, but an integration-time-dependent radiometric calibration framework coupled with representative spectral-emissivity transfer under clearly stated applicability conditions. Its central element is a three-parameter radiometric calibration model in which camera integration time is explicitly included, so that radiance conversion can be performed across the experimentally calibrated integration-time range without repeating a separate fixed-exposure calibration for each setting. Multiwavelength spectral radiance is used to jointly retrieve temperature and a continuous, second-order polynomial emissivity function with a genetic algorithm serving as the global optimizer. The emissivity function obtained from a representative spectral sampling region is then transferred to the imaging model for pixelwise temperature inversion; this step assumes that the material and surface state are sufficiently uniform over the region to which the function is applied. The method is examined using steady-state tungsten–halogen-lamp measurements with nominal color temperatures of 2200–2800 K and a transient laser-heated 316L stainless-steel case. Agreement with a Wien-based estimate is used as an internal spectral-consistency check rather than as an independent traceable accuracy validation. In the transient case, the retrieved spectral-field-of-view temperature increased from 2311.9 to 2398.5 K over 50–60 s, and the reconstructed images reproduced the corresponding increase in the central high-temperature region. The present results demonstrate the feasibility of coupling integration-time-dependent calibration with measured spectral-emissivity transfer for two-dimensional temperature reconstruction, while the achievable absolute accuracy remains subject to detector linearity, emissivity-model validity, spatial emissivity uniformity, radiometric calibration, and independent reference validation.

## 1. Introduction

Measurement of high-temperature fields is important in scientific and engineering applications including explosion diagnostics, laser–matter interaction analysis, combustion monitoring, metallurgical process control, laser material processing, and aerospace thermal-protection testing [1,2,3,4]. Accurate determination of spatiotemporal temperature distributions under extreme thermal conditions supports analysis of the underlying physical mechanisms, process control, and safety assessment.

Conventional contact thermometry, such as thermocouples and resistance temperature detectors, is fundamentally limited in these applications. Its finite response time prevents the capture of rapid thermal transients, while the required physical contact is intrusive or impractical in chemically reactive or physically inaccessible high-temperature regions [5,6]. Consequently, non-contact thermometry has become a major focus of research.

A variety of non-contact optical thermometry techniques have been proposed, including laser-induced fluorescence (LIF) thermometry [7], Raman-scattering thermometry [8], and thermographic-phosphor thermometry [9]. Although these techniques can provide high accuracy or high spatial resolution under suitable conditions, they generally require specific optical access, excitation, or calibration arrangements, and their applicability depends on the measurement environment. For two-dimensional high-temperature-field measurements, commonly used optical approaches include multispectral or color imaging, monochrome imaging combined with spectral information, and infrared thermography. Silicon imaging detectors are attractive because they are widely available and provide high spatial resolution in the visible–near-infrared (VIS–NIR) range, where radiation from sufficiently hot targets can be detected. The most appropriate technique therefore depends on the metrological objective: traceable single-point measurements favor calibrated radiation thermometry referenced to the International Temperature Scale of 1990 (ITS-90), spectral characterization favors multiwavelength thermometry, and spatial-field measurements favor imaging or spectral–image fusion approaches [5,10].

Substantial research has advanced the development of CCD pyrometry. Fu et al. [11] proposed a three-color optical CCD pyrometer that improved measurement stability through multispectral-channel fusion. Jegou et al. [12] developed a two-color melt-pool thermometry method based on a single RGB camera and applied it to additive manufacturing, demonstrating that CCD-based two-color thermometry can provide effective temperature monitoring when the emissivity ratio is well behaved. Li et al. [13] systematically investigated the effects of internal modulation parameters on the temperature-calculation performance of a matrix-search CCD pyrometer, revealing the influence of camera settings such as gain and gamma correction on measurement accuracy. Yu et al. [14] proposed a CCD thermometry method that does not require calibration of the channel proportionality coefficient, thereby substantially simplifying the calibration procedure through the solution of a set of integral equations. Kikuchi et al. [15] used thermal-radiation imaging with a CCD camera to estimate the temporal temperature distribution during blue-laser welding of copper, demonstrating the feasibility of CCD thermometry in laser-processing applications. Li et al. [16] developed a matrix-search-based color CCD method for high-temperature measurement that partially mitigates the effects of calibration parameters and variations in spectral emissivity. Batchuluun et al. [17] applied deep learning to thermal-image reconstruction, while Du et al. [18] proposed a spectral-adaptive-correction method for temperature inversion during laser ablation using a CMOS detector, further extending the application of CMOS detectors in multispectral thermometry.

Despite these advances, a fundamental challenge remains: an imaging detector measures radiation integrated over its spectral response, whereas conversion from radiance to temperature requires knowledge of the target spectral emissivity. Many imaging methods assume gray-body behavior, i.e., wavelength-independent emissivity, or use two-color ratios that reduce emissivity dependence only when the emissivity ratio at the selected wavelengths is known or sufficiently close to unity [19]. These assumptions can be violated in nonuniform high-temperature fields such as flames, laser-heated surfaces, and other processes in which emissivity varies with wavelength, temperature, oxidation state, or phase. Pelzmann et al. [20] showed that spectral-emissivity variation can materially affect two-color thermometry, while Zhu et al. [21] demonstrated that temperature retrieval with unknown emissivity remains sensitive to the adopted emissivity model. Thus, CCD-based thermometry remains useful, but its quantitative accuracy depends strongly on how emissivity is represented or measured.

The limitation of a gray-body assumption can also be expressed through the local sensitivity of radiation temperature to emissivity. Under a Wien-type approximation, a small emissivity mismatch produces an approximate relative temperature bias *ΔT*/*T* ≈ −(*λT*/*c*_2_)(*Δε*/*ε*), showing that even a moderate spectral-emissivity error can translate into a non-negligible temperature bias at high temperature. Existing strategies therefore include two-color methods with measured or modeled emissivity ratios [19,20,21], multiwavelength thermometry with parameterized emissivity [22,23,24,25,26,27,28,29,30,31], direct spectroscopic determination of wavelength-dependent emissivity [32,33], spectral–image fusion [33,34,35,36,37], and emissivity estimation through complementary reflectance measurements.

To address the emissivity problem more fundamentally, multiwavelength, or spectral, thermometry has been extensively developed [38,39]. This approach acquires radiance measurements at multiple wavelengths and simultaneously retrieves temperature and spectral emissivity by assuming that the wavelength dependence of emissivity follows a parameterized form. Common emissivity models include polynomial, exponential, and logarithmic forms. Xing et al. [26] proposed an algorithm that adaptively determines the emissivity-model form during data processing without requiring it to be specified a priori. Taunay and Choueiri [27] introduced a robust cross-validation framework for selecting the optimal emissivity model from multiple candidates, thereby improving measurement reliability. Liu and Zheng [28] demonstrated the reconstruction of wavelength-dependent emissivity distributions using multiwavelength radiation thermometry. Wang et al. [29] developed an advanced multispectral thermometry method based on an improved light-spectrum optimizer and achieved reliable temperature and emissivity retrieval through a nature-inspired optimization algorithm. Yuan et al. [30] established a high-precision temperature-calibration method for spectral-emissivity measurements at low temperatures, providing a sound metrological basis for spectral radiation thermometry. In a noteworthy recent development, He et al. [31] proposed dispersive metalens thermometry, in which the spectral components of thermal radiation are spatially separated across a detector array to enable high-accuracy, single-shot multispectral temperature measurement, highlighting the increasing technological importance of spectral thermometry.

Integrating spectral thermometry with CCD imaging is a natural development that combines the spectral richness of multiwavelength measurements with the spatial resolution of CCD arrays. The authors’ group [34] previously proposed a spectral–image fusion method for reconstructing temperature distributions in deflagration fields, enabling quantitative characterization of nonuniform temperature fields through joint inversion of spectral information and CCD images. Wang et al. [35] recently proposed an adaptive multispectral radiation-thermometry algorithm that performs multispectral temperature measurements at multiple spatial locations and fuses the results with three-dimensional imagery to reconstruct a three-dimensional temperature distribution. Lin et al. [32] developed a spectroscopically determined emissivity correction for color-ratio thermometry and applied it to hydrocarbon-flame temperature measurement. By replacing the gray-body assumption with measured spectral emissivity, their temperature results agreed closely with thermocouple measurements. Yan et al. [33] combined spectral and image thermometry for biomass combustion in a laboratory-scale furnace. Using a second-order polynomial to describe nongray emissivity, they achieved accurate temperature and emissivity estimates under practical combustion conditions. Pan et al. [36] selected characteristic spectral lines by laser-induced breakdown spectroscopy (LIBS) and constructed a dual-CMOS imaging system for high-accuracy two-dimensional temperature-field measurement of nitrocellulose flames. In laser-related applications, Schulte et al. [37] demonstrated in situ, high-speed near-infrared imaging during laser sintering of nanoparticles and obtained temporally and spatially resolved temperature maps using a CCD camera coupled to an optical microscope, further confirming the importance of CCD thermometry in laser processing.

Despite the progress of combined spectral–imaging thermometry, two practical limitations remain relevant to the present work. First, many imaging radiometric calibrations are performed at a fixed integration time, so a change in exposure requires a new calibration relationship or an additional correction [32,33]. Second, spectral emissivity is usually measured only over one or several representative regions, whereas imaging requires a temperature solution at every valid pixel. Existing studies have commonly transferred a representative emissivity to the full field when the surface is assumed spatially uniform [32,33,34], used multiple spatial spectral measurements with interpolation or regional processing [35,36], or introduced a parameterized emissivity model that can be evaluated continuously in wavelength. The remaining difficulty is therefore not that each pixel lacks an independently measured emissivity value, but that the transfer from discrete spectral sampling to image-wide temperature inversion is valid only when its spatial-representativeness assumptions are satisfied.

Against this background, the contribution of the present study is an integrated calibration–emissivity-transfer framework rather than a new multispectral principle or a new optimization algorithm. The first and central element is a three-parameter camera radiometric model that explicitly includes integration time and is intended for use within the experimentally established linear-response and calibration ranges. The second element is a temperature–emissivity joint inversion in which a genetic algorithm is used as a global optimizer and a low-order polynomial provides a continuous wavelength-dependent emissivity function from representative spectral measurements. The third element is the transfer of this measured emissivity function into the imaging equation for pixelwise temperature inversion, together with explicit discussion of the assumptions governing Lambertian behavior, temperature dependence of emissivity, spatial representativeness, polynomial-model validity, and uncertainty propagation. Relative to Refs. [37,38,39,40,41], the distinction therefore lies primarily in the integration-time-dependent calibration architecture and in how the representative spectral emissivity is coupled to the image inversion under stated applicability limits.

The framework is examined in two experiments: a steady-state tungsten–halogen-lamp experiment with nominal color temperatures of 2200–2800 K and a transient experiment in which 316L stainless steel is laser heated. In the steady-state experiment, comparison with a Wien-based temperature estimate is used to assess internal consistency between two spectral processing routes; because both originate from the measured radiation spectrum, this comparison is not treated as an independent traceable temperature validation. The transient experiment is used to demonstrate the temporal and spatial behavior recovered by the coupled spectral–image procedure.

The remainder of this paper is organized as follows. Section 2 presents the fundamentals of multiwavelength spectral thermometry and the proposed emissivity-corrected CCD thermometry model. Section 3 describes the experimental setup, the CCD radiometric calibration procedure, and the experimental results under steady-state and transient conditions. Section 4 summarizes the findings and outlines future research directions.

## 2. Theoretical Basis and Methodology

The proposed joint thermometry method comprises two coupled stages. In the first stage, a fiber-optic spectrometer acquires radiation from a representative target region, and quantitative temperature–emissivity analysis is performed over the calibrated 500–760 nm band used in this study. After radiometric calibration converts the measurement into spectral radiance, a genetic algorithm is used to jointly retrieve the target temperature and the coefficients of a continuous emissivity polynomial. In the second stage, a monochrome imaging camera synchronously acquires a two-dimensional gray-level image of the same scene. The representative emissivity function obtained from the spectral stage is then incorporated into the image radiometric model, and temperature is solved pixel by pixel. The transfer does not constitute an independent emissivity measurement at every pixel and is valid only when the spectral sampling region is representative of the image region to which the function is applied.

### 2.1. Principles of Multispectral Thermometry

#### 2.1.1. Fundamentals of Radiation Thermometry

According to Planck’s law, the spectral radiant exitance of a blackbody at absolute temperature T and wavelength λ is

(1) $$M_{b}(\lambda ,T)=\frac{C_{1}}{\lambda^{5}}\frac{1}{e^{C_{2}/(\lambda T)}-1}$$

where $C_{1}=2\pi hc^{2}=3.7418\times 10^{-16}\;W\cdot m^{2}$ is the first radiation constant, $C_{2}=hc/k=1.4388\times 10^{-2}\;m\cdot K$ is the second radiation constant, $h=6.6261\times 10^{-34}\;J\cdot s$ is Planck’s constant, $k=1.3806\times 10^{-23}\;J\cdot K^{-1}$ is the Boltzmann constant, and $c=2.9979\times 10^{8}\;m\cdot s^{-1}$ is the speed of light in vacuum.

For a real object, the spectral emissivity ε(λ) is introduced to characterize its radiative capability relative to that of a blackbody at the same temperature. In most engineering radiation-thermometry applications, a high-temperature solid surface can be approximated as a diffuse radiator, or Lambertian surface, whose radiance is independent of direction. Under this assumption, the relationship between the spectral radiance L(λ,T) and radiant exitance is L=M/π, and therefore

(2) $$L(\lambda ,T)=\varepsilon (\lambda )\frac{C_{1}}{\pi \lambda^{5}}\frac{1}{e^{C_{2}/(\lambda T)}-1}$$

Within the temperature (T∈[1500,3000] K) and wavelength (λ∈[400,1000] nm) ranges considered in this study, the condition $\lambda T\ll C_{2}$ is always satisfied; therefore, the radiance can be accurately approximated using Wien’s radiation law as

(3) $$L(\lambda ,T)\approx \varepsilon (\lambda )\frac{C_{1}}{\pi \lambda^{5}}e^{-C_{2}/(\lambda T)}$$

#### 2.1.2. Polynomial Emissivity Model

Multiwavelength thermometry requires spectral emissivity ε(λ) to be represented by a low-dimensional function so that temperature and emissivity can be retrieved from a finite set of spectral measurements. In the 500–760 nm analysis band used here, the measured spectra considered in the steady-state and transient experiments are smooth and do not contain pronounced narrowband features. A low-order polynomial is therefore adopted as a regularized representation of this slowly varying wavelength dependence. This choice is a modeling assumption used to stabilize the inverse problem rather than an innovation of the present study, and its suitability must be reassessed for materials with strong narrowband absorption or emission features.

Polynomial order is controlled using the Akaike information criterion (AIC):

AIC=Nln(RSS/N)+2K

where *N* is the number of valid wavelength channels, RSS is the residual sum of squares, and K is the number of fitted parameters. Reference high-temperature emissivity data and the steady-state residual comparison in Section 3 support the use of a second-order polynomial as a practical balance between residual reduction and model complexity for smooth VIS–NIR emissivity. The available archived data do not contain a complete, reproducible set of material-specific first-, second-, and third-order AIC/BIC values and temperature sensitivities for both tungsten and 316L; therefore, no such material-specific numerical table is claimed here. A systematic order-sensitivity analysis using the same optimization settings is identified as a required future validation.

Emissivity-model performance is material-dependent. Previous multiwavelength radiation-thermometry studies have shown that polynomial and log-linear models can perform differently for different metals and alloys [42,43,44,45,46]. Accordingly, the second-order polynomial adopted here should be interpreted as a compact representation suitable for the smooth spectra observed in the present analysis band, not as a universal emissivity law for all materials.

On this basis, the spectral emissivity used in the present inversion is represented by the following second-order polynomial:

(4) $$\varepsilon (\lambda )=a_{0}+a_{1}\lambda +a_{2}\lambda^{2}$$

where $a_{0},a_{1},a_{2}$ are the polynomial coefficients to be determined. The emissivity must satisfy the physical constraint

(5) 0<ε(λ)<1, ∀λ∈[400,1000] nm

This constraint is used as a feasibility criterion in the GA optimization. Substituting Equation (4) into Equation (2), with the Wien approximation in Equation (3), gives the theoretical radiance at the ith wavelength channel $\lambda_{i}$ as

(6) $$L(\lambda_{i},T,a)=(a_{0}+a_{1}\lambda_{i}+a_{2}\lambda_{i}^{2})\cdot \frac{C_{1}}{\pi \lambda_{i}^{5}}e^{-C_{2}/(\lambda_{i}T)}$$

where $a=[a_{0},a_{1},a_{2}{]}^{T}$ is the emissivity-coefficient vector.

#### 2.1.3. Temperature–Emissivity Joint Inversion Procedure

The spectrometer provides calibrated radiance values at N wavelength channels. For the second-order emissivity model, the joint inverse problem contains four unknowns: the temperature T and the three emissivity coefficients a_0_, a_1_, and a_2_. Because the number of wavelength channels is much larger than four, the data provide an overdetermined fit; however, temperature and emissivity remain coupled nonlinearly and can be ill-conditioned under finite signal-to-noise conditions. The polynomial constraint reduces the emissivity from an unrestricted function to three coefficients and thereby regularizes the temperature–emissivity separation problem.

Constraining ε(λ) to a second-order polynomial, as in Equation (4), regularizes the ill-posed problem by reducing the infinite-dimensional function-estimation problem to a finite-dimensional problem with three coefficients and by introducing prior information on the known slow variation of ε(λ) with wavelength. The regularized parameter space is $R^{4}$. If the measurement noise is sufficiently low and the true form of ε(λ) is adequately approximated by a second-order polynomial, then $\left\{T,a_{0},a_{1},a_{2}\right\}$ is identifiable, i.e., a unique optimum exists.

The inverse problem is formulated as a constrained nonlinear least-squares problem. A genetic algorithm (GA) is used here as the global optimizer because the coupled temperature–emissivity objective can be sensitive to initialization and may contain multiple local minima. The GA is not introduced as a new algorithm in this work; it is the numerical tool selected to solve the proposed joint-inversion procedure. The temperature obtained from the spectral stage is subsequently used as the initial estimate for the pixelwise Newton–Raphson solution in the imaging stage.

GA parameter settings. The search-space dimension is D=4. The population size is $N_{pop}=300$, the maximum number of generations is M=500, the crossover probability is $P_{c}=0.8$, and the initial mutation probability is $P_{m}^{\left(0\right)}=0.05$. The selection operator combines tournament selection (tournament size = 3) with elitism; the best 5% of individuals in each generation are passed directly to the next generation to prevent high-quality solutions from being destroyed by crossover or mutation. Single-point and uniform crossover are alternated, with single-point crossover used in odd-numbered generations and uniform crossover in even-numbered generations, thereby balancing preservation of local gene segments with thorough mixing of global gene positions. A nonuniform mutation operator is employed: at generation g, the mutation step decreases according to $\Delta (g)=\Delta_{0}\cdot (1-g/M{)}^{\beta }$ with β=3, allowing broad exploration of the solution space during the early stage and progressively finer exploitation during the later stage.

Fitness function. The fitness function F(x) is constructed from the weighted sum of squared residuals over all wavelengths:

(7) $$F(x)=\sum_{i=1}^{N}w_{i}{\left[L_{meas}(\lambda_{i})-L(\lambda_{i},T,a)\right]}^{2}$$

where $x=(T,a_{0},a_{1},a_{2})$, and the weighting coefficient $w_{i}$ is taken as the normalized spectrometer response:

(8) $$w_{i}=\frac{S(\lambda_{i})}{\sum_{j=1}^{N}S(\lambda_{j})}$$

where $S(\lambda_{i})$ is the raw spectrometer signal at the ith channel. Using the measured signal amplitude as the weighting factor naturally assigns greater residual weight to channels with higher signal-to-noise ratios (SNRs), thereby suppressing the adverse influence of noise in low-response regions, such as the short- and long-wavelength ends where the detector quantum efficiency decreases. This weighting strategy assumes that the spectrometer response $S(\lambda_{i})$ has been corrected for relative spectral responsivity, i.e., that the wavelength dependence of the system response has been removed. If $S(\lambda_{i})$ contains substantial uncorrected system-response variation, the weights $w_{i}$ may amplify systematic bias. In practice, relative spectral-response correction is completed during radiometric calibration to ensure that $S(\lambda_{i})$ faithfully represents the relative spectral distribution of the incident radiance.

The GA terminates when either the prescribed maximum number of generations is reached, or the relative change in the best fitness value remains below 10^−5^ for 50 consecutive generations. Because the GA is stochastic, it is executed repeatedly for each dataset, and the run with the lowest fitness is retained. The convergence-rate values reported in Table 1 denote the fraction of independent runs that satisfy the fitness-stability criterion and the prescribed solution tolerance; this numerical repeatability should not be interpreted as an experimental temperature-accuracy metric.

The resulting temperature and emissivity coefficients define a continuous representative emissivity function that is transferred to the subsequent image-thermometry stage for pixelwise temperature inversion. The solved quantity at each image pixel is temperature, whereas emissivity is obtained from the representative spectral sampling region and is not independently measured for every pixel. Therefore, the validity of this transfer depends on the assumption that the emissivity distribution within the applied imaging region is sufficiently uniform or that its spatial variation is limited.

#### 2.1.4. Numerical Validation of the Inversion Method

Synthetic-data experiments were conducted to evaluate the identifiability and accuracy of the GA inversion. The basic procedure was to prescribe known ground-truth parameters $\left(T^{*},a_{0}^{*},a_{1}^{*},a_{2}^{*}\right)$, calculate a noise-free theoretical radiance spectrum using Equation (6), add Gaussian noise at different levels to simulate actual measurements, perform the GA inversion, and compare the retrieved parameters with the known ground truth.

Experimental design. The ground-truth parameters were selected to represent a typical high-temperature silicon-carbide (SiC) radiation scenario: $T^{*}=2000\;K$, with emissivity coefficients $a_{0}^{*}=0.92$, $a_{1}^{*}=-3.5\times 10^{-4}\;nm^{-1}$, and $a_{2}^{*}=2.0\times 10^{-7}\;nm^{-2}$. Over 400–1000 nm, these parameters yield an emissivity of approximately 0.77–0.81, consistent with the typical experimental range of high-temperature SiC emissivity in the VIS–NIR region (0.80–0.95, as reported for reference SiC materials in the TPRC handbook by Touloukian and DeWitt [42] and Neuer and Jaroma-Weiland [47]). The decreasing spectral trend is also qualitatively consistent with the Drude free-carrier absorption model and the Hagen–Rubens relation. The quadratic coefficient $a_{2}^{*}>0$ provides only a slight positive-curvature correction over the fitting interval; its value is determined by statistical optimality rather than by an independent physical mechanism, which should be recognized when discussing model applicability.

The wavelength range was 400–1000 nm with a 1 nm interval, giving 601 channels and covering the actual operating range of the spectrometer. Wavelength-independent additive Gaussian noise was introduced to represent a measurement scenario dominated by detector dark noise and readout noise. Eight standard-deviation levels, $\sigma_{n}=0.1\%,0.2\%,0.5\%,1\%,2\%,5\%,10\%,20\%$, were considered as percentages of the peak noise-free radiance, spanning conditions from an ideal laboratory environment to a harsh industrial setting. Under this definition, the actual SNR at the spectral wings is lower than that at the peak. This effect contributes appreciably to the inversion error when $\sigma_{n}\geq 5\%$, and is used below to explain the nonlinear increase in error.

For each noise level, 100 independent noisy synthetic spectra were generated by Monte Carlo simulation. The GA was independently executed K=10 times for each spectrum, and the best result was retained.

The following evaluation metrics were used:

- Relative bias of the retrieved temperature: $\delta_{T}=|\hat{T}-T^{*}|/T^{*}$;
- Root-mean-square error of the retrieved emissivity: ${RMSE}_{\varepsilon }=\sqrt{\frac{1}{N}\sum_{i=1}^{N}[\hat{\varepsilon }(\lambda_{i})-\varepsilon^{*}(\lambda_{i}){]}^{2}}$;
- Mean ($\bar{T},{\bar{a}}_{k}$) and coefficient of variation (CV = standard deviation/mean) of each retrieved parameter.

Validation results for synthetic data representing a typical SiC spectral scenario ($T^{*}=2000\;K$) are summarized in Table 1 for the different noise levels.

Under low-noise conditions ($\sigma_{n}\leq 1\%$), the GA yielded a temperature bias below 0.2%, an emissivity RMSE on the order of $10^{-3}$, and a convergence rate close to 100%, confirming both the identifiability of T–ε separation and the high inversion accuracy under typical laboratory conditions. Even under moderate noise ($\sigma_{n}=5\%$), the temperature bias remained below 1% (approximately 13 K), while the emissivity RMSE was approximately 0.018, satisfying the accuracy requirements of most engineering thermometry applications. At a noise level of 20%, the temperature bias increased to approximately 3% (approximately 56 K), and the convergence rate decreased to 78.6%, indicating a substantial loss of inversion reliability under such extreme noise.

The numerical validation yielded the following principal conclusions:

1. Identifiability: under normal SNR conditions, the second-order polynomial constraint is sufficient to regularize the T–ε separation problem, and the four-parameter joint inversion has a unique identifiable optimum;
2. Accuracy baseline: under typical laboratory conditions with noise below 1%, the relative error of the retrieved temperature can be maintained below 0.2%;
3. SNR threshold: when $\sigma_{n}>10\%$, inversion reliability decreases markedly. In practical applications, the spectral-signal SNR should be maintained above 20 dB, corresponding to noise below 10%, to ensure reliable inversion;
4. GA stability: the statistics from K=10 independent runs show good run-to-run consistency under $\sigma_{n}\leq 5\%$ conditions, with CV < 1%.

### 2.2. Principles of Emissivity-Corrected CCD Thermometry

#### 2.2.1. Physical Link from Source Radiance to Pixel Gray Level

After the temperature and emissivity have been jointly retrieved from a single-point spectrum, the second stage transfers the resulting emissivity information to the two-dimensional CCD imaging system to enable full-field temperature measurement.

Definition and basic relationship of radiance. Consider a surface element of the target at temperature T, whose spectral radiance L(λ,T) is given by Equation (2). Radiance L is defined as the radiant flux emitted per unit projected area and per unit solid angle, with units of $W\cdot m^{-2}\cdot sr^{-1}$. The radiant flux emitted by source-surface element dS at wavelength λ toward the camera lens is

(9) $$\Phi (\lambda ,T)=L(\lambda ,T)\cdot dS\cdot \Omega_{r}$$

where dS is the area of the source-surface element, determined by the object–image relationship of the imaging system and the pixel size, and $\Omega_{r}$ is the solid angle subtended by the lens at surface element dS.

**Figure 1:**
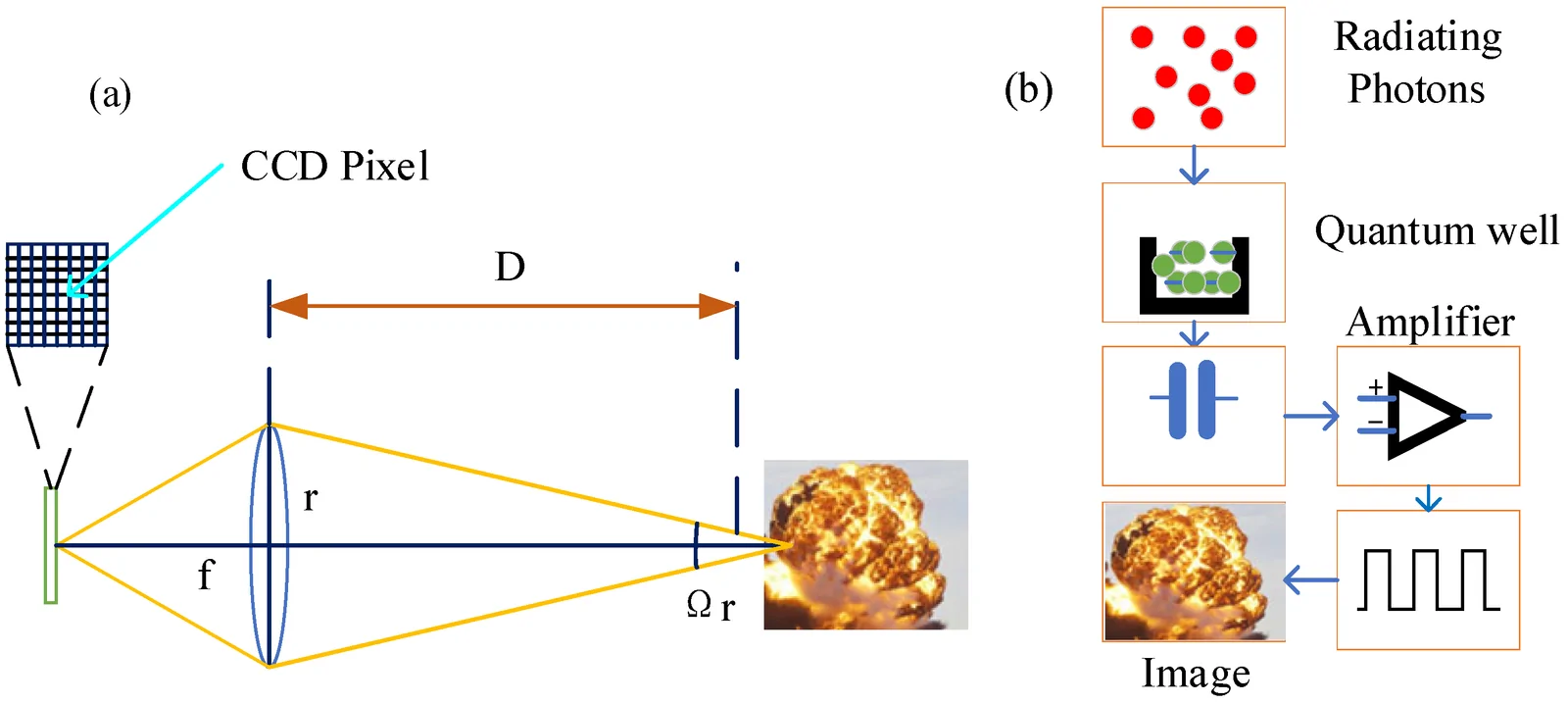
Radiometric calibration model: (**a**) physical imaging path; (**b**) photoelectric conversion process.

Figure 1a illustrates the imaging path from a source-surface element to a CCD pixel. For an imaging system with pixel size P, focal length f, and object distance D, the object-space area corresponding to a single pixel is obtained from the thin-lens equation and similar-triangle relationships as

(10) $$dS={\left(\frac{P\cdot D}{f}\right)}^{2}$$

Note that Equation (10) is strictly valid when the surface element lies on the optical axis, and the object distance D is much greater than the image distance (D≫f). For an off-axis field of view, the surface-element area and projection effect introduce an additional cos θ factor. Under the far-field imaging conditions used in this study (D≫f, field angle < 5°), the error introduced by this paraxial approximation is below 1%.

When the lens radius r is much smaller than the object distance D, the solid angle subtended by the lens at the surface element is

(11) $$\Omega_{r}=\frac{S}{D^{2}+r^{2}}=\frac{\pi r^{2}}{D^{2}+r^{2}}$$

where $S=\pi r^{2}$ is the lens-aperture area. Equation (11) is derived from the solid-angle definition $\Omega =A/R^{2}$ and is strictly applicable to an on-axis surface element whose distance from the lens center is $\sqrt{D^{2}+r^{2}}$. At large off-axis field angles, the projected aperture area viewed from the oblique surface element decreases, and the actual solid angle is therefore slightly smaller than that calculated from Equation (11). Under the imaging configuration used here (D≫r, field angle < 5°), the paraxial condition is satisfied, and the solid-angle approximation error is negligible.

Substitution of Equations (10) and (11) into Equation (9) gives the radiant flux received by a single pixel:

(12) $$\Phi (\lambda ,T)=L(\lambda ,T)\cdot \frac{P^{2}D^{2}}{f^{2}}\cdot \frac{\pi r^{2}}{D^{2}+r^{2}}=L(\lambda ,T)\cdot \frac{\pi r^{2}P^{2}}{f^{2}}\cdot \frac{D^{2}}{D^{2}+r^{2}}$$

Under far-field imaging conditions (D≫r), $\frac{D^{2}}{D^{2}+r^{2}}\approx 1$, and Equation (12) simplifies to

(13) $$\Phi (\lambda ,T)=\frac{\pi r^{2}P^{2}}{f^{2}}\cdot L(\lambda ,T)=R_{O}\cdot L(\lambda ,T)$$

where $R_{O}=\pi r^{2}P^{2}/f^{2}$ is the radiant-flux collection factor of the optical system. During measurement, the camera parameters P,f,r and object distance D remain constant; therefore, $R_{O}$ can be treated as a system constant independent of the incident radiation. Equation (13) shows that, under far-field conditions, the radiant flux Φ received by a single pixel is linearly proportional to the radiance L.

Photoelectric conversion and gray-level output. As shown in Figure 1b, a CCD pixel converts incident radiant energy into an electrical signal. If the radiant flux incident on a pixel per unit time is Φ(λ,T), the corresponding photon flux is Φ(λ,T)/(hν), and the number of photons absorbed by the pixel per unit time is

(14) $$N=\frac{\Phi (\lambda ,T)}{h\nu }=\frac{\Phi (\lambda ,T)\cdot \lambda }{hc}$$

Photons are absorbed in the silicon photodiode and converted into electron–hole pairs. The quantum efficiency η is defined as the ratio of the number of generated photoelectrons to the number of incident photons:

(15) $$\eta (\lambda )=\frac{n_{e}}{N}$$

where $n_{e}$ is the charge generated by the optical signal. The quantum efficiency η(λ) is wavelength-dependent. For a typical front-illuminated silicon CCD, η reaches a maximum of approximately 40–60% over 500–700 nm and gradually decreases toward both spectral ends. This wavelength dependence must be included in the calibration and subsequent full-band integration.

The charge accumulated in a pixel is converted through charge-to-voltage conversion, linear amplification, and analog-to-digital conversion (ADC), and is output as the digital gray level [41]:

(16) $$G=K_{d}\cdot n_{e}\cdot t_{int}+N_{dark}\cdot t_{int}+N_{read}$$

where the detector gain describes the conversion from photoinduced charge to digital output, and t is the integration time. The integration-time-independent offset in this expression should be interpreted primarily as the electronic readout baseline associated with the charge-to-voltage conversion, amplification, and A/D conversion chain. Thermally generated dark charge is not strictly integration-time-independent; residual dark charge accumulates during exposure and is therefore treated together with other time-dependent background contributions in the empirical calibration model below. This distinction indicates that the constant term represents the readout baseline, while exposure-dependent contributions include residual dark-current accumulation, environmental background radiation, and optical stray radiation. This treatment does not imply that these contributions originate from the same physical mechanism but indicates that they exhibit similar dependence on integration time within the operating range considered in this study.

The on-chip black-level correction (BLC) function implemented in the onsemi PYTHON300 sensor (onsemi, Scottsdale, AZ, USA) should be distinguished from dark-current correction. The BLC operation primarily compensates for fixed-pattern electronic offsets introduced by the sensor readout chain, typically using optical black pixels or internal reference signals to establish the zero-signal baseline. BLC can therefore effectively reduce the fixed-pattern component of the electronic baseline, but it does not eliminate thermally generated dark charge accumulated during exposure, which remains integration-time dependent and is included in the exposure-dependent background term of the calibration model. In this study, the BLC function was enabled during image acquisition to minimize the influence of readout-level baseline drift and improve the stability of radiometric response calibration.

Combining Equations (13)–(16) gives the complete response relationship between radiance and pixel gray level:

(17) $$\begin{array}{cc} G(\lambda ,T,t_{int}) & =K_{d}\cdot \eta (\lambda )\cdot \frac{\lambda }{hc}\cdot R_{O}\cdot L(\lambda ,T)\cdot t_{int}+G_{read}\\ & =R_{\lambda }\cdot L(\lambda ,T)\cdot t_{int}+G_{read} \end{array}$$

Combining the optical collection and photoelectric-conversion relations gives the radiance-to-gray-level response. The important feature is that the radiation-dependent signal scales with integration time while the camera operates in its experimentally determined linear-response range. The measured output also contains an electronic baseline and exposure-dependent background contributions, which are treated separately in the three-parameter calibration model of Section 2.2.2.

Integration-time constraint. The linear relationship assumed in the model is valid only within the detector range established experimentally. At very low signal levels, readout and background noise become a large fraction of the measured gray level; near saturation or the full-well limit, the gray-level–radiance relationship becomes nonlinear and signal clipping may occur. Integration time must therefore be chosen so that the pixels used for thermometry remain sufficiently above the noise floor and below saturation. The present model should be regarded as an interpolation model within the tested linear and calibration ranges rather than as an unrestricted extrapolation to arbitrary exposure settings.

Full-band integration and long-wavelength response. A CCD detector records the accumulated radiation over the entire silicon-response range, approximately 350–1100 nm. Integrating Equation (17) over the detector operating band $\left[\lambda_{min},\lambda_{max}\right]$ gives the total pixel gray level:

(18) $$G\left(t_{int}\right)=\int_{\lambda_{min}}^{\lambda_{max}}R_{\lambda }\cdot \varepsilon \left(\lambda \right)\cdot \frac{C_{1}}{\pi \lambda^{5}}\frac{1}{e^{\frac{C_{2}}{\lambda T}}-1}\;d\lambda \cdot t_{int}+G_{read}$$

where the emissivity $\varepsilon (\lambda_{i})$ is calculated by substituting the coefficients $\left({\hat{a}}_{0},{\hat{a}}_{1},{\hat{a}}_{2}\right)$ retrieved by the GA in Section 2.1 into Equation (4). In practical processing, the integral in Equation (18) is approximated by a discrete sum over the spectrometer wavelength channels.

#### 2.2.2. Radiometric Calibration Model

Equation (18) can be represented using three effective response parameters that characterize the radiance-dependent signal, the exposure-time-dependent contribution, and the integration-time-independent electronic baseline. This parameterization is intended to describe the observed camera response over the calibrated linear range without assigning each fitted coefficient to a single, independently measured physical mechanism.

(19) $$G=t_{int}\cdot \bar{R}\cdot \bar{L}(T)+t_{int}\cdot G_{out}+G_{in}$$

In the three-parameter form, the radiance-dependent coefficient represents the camera response gain per unit integration time. The time-dependent offset term includes contributions that can accumulate approximately linearly during exposure, such as residual dark charge, environmental background radiation, and optical stray radiation. The constant term is defined as the electronic readout baseline remaining when integration time is extrapolated toward zero, including fixed offsets in the conversion, amplification, and A/D readout chain; it is not interpreted as a pure dark-current coefficient.

The onsemi PYTHON300 sensor used in this study provides on-chip black-level correction (BLC). BLC reduces fixed readout-level offsets but does not imply complete removal of thermally generated dark current. A strict separation of detector dark accumulation from optical-background and stray-radiation contributions would require dedicated measurements: dark frames with the lens blocked at multiple integration times, background frames with the optical path open but without target radiation, and measurements at multiple known radiance levels. The present archived dataset was not designed for this decomposition, so separate dark-current and stray-light coefficients are not reported.

For an ideal blackbody reference, the calibration relation can be written in the simplified form of Equation (20). In the present experiment, however, the tungsten–halogen source is not assumed to be an ideal blackbody over the full VIS–NIR range. Quantitative processing uses the calibrated spectral-radiance information over the 500–760 nm analysis band; the nominal lamp color temperature is used only to label the source operating condition and is not substituted as a thermodynamic-temperature ground truth.

(20) $$G(T_{cal},t_{int})=t_{int}\cdot \bar{R}\cdot {\bar{L}}_{b}(T_{cal})+t_{int}\cdot G_{out}+G_{in}$$

The three parameters are identified from measurements at multiple known radiance levels and multiple integration times. At a fixed integration time, variation in the reference radiance constrains the response slope and combined offset; repeating the measurement at several integration times then separates the exposure-dependent term from the zero-time electronic baseline. The fit is therefore meaningful only over the radiance and integration-time domain represented by the calibration data. Additional integration times withheld from fitting would provide a stronger independent test of interpolation performance, but the original archived data do not support such a leave-one-integration-time-out validation.

#### 2.2.3. Pixelwise Temperature Inversion Using a Representative Spectral Emissivity Function

Once the calibration parameters have been determined, the gray level of each valid image pixel is converted to band-equivalent radiance after accounting for the fitted exposure-dependent background term and the electronic readout baseline.

(21) $$G_{corr}^{\left(j,k\right)}=G^{\left(j,k\right)}-t_{int}\cdot G_{out}-G_{in}=t_{int}\cdot \bar{R}\cdot \bar{L}(T_{j,k};\hat{\varepsilon })$$

where $\bar{L}(T_{j,k};\hat{\varepsilon })$ is the band-equivalent radiance calculated using the retrieved emissivity polynomial $\hat{\varepsilon }(\lambda )={\hat{a}}_{0}+{\hat{a}}_{1}\lambda +{\hat{a}}_{2}\lambda^{2}$, expressed as

(22) $$\bar{L}(T_{j,k};\hat{\varepsilon })=\frac{\sum_{i=1}^{N}R(\lambda_{i})\cdot \hat{\varepsilon }(\lambda_{i})\cdot L_{b}(\lambda_{i},T_{j,k})\cdot \Delta \lambda }{\sum_{i=1}^{N}R(\lambda_{i})\cdot \Delta \lambda }$$

where $R(\lambda_{i})$ is the discrete spectral responsivity at each wavelength channel, obtained from calibration, and $L_{b}(\lambda_{i},T_{j,k})$ is calculated using the exact form of Planck’s law in Equation (1) divided by π. The Wien approximation is not used in the pixelwise iterative inversion to avoid accumulation of approximation error.

After the emissivity polynomial $\hat{\varepsilon }(\lambda )$ and temperature $\hat{T}$ have been retrieved from the single-point spectrum using Equations (4) and (7), $\hat{\varepsilon }(\lambda )$ is substituted into Equation (21). A scalar nonlinear equation is then solved for each pixel (j,k) to obtain the corresponding temperature $T_{j,k}$. The solution is obtained using a one-dimensional Newton–Raphson iteration:

(23) $$T_{j,k}^{\left(m+1\right)}=T_{j,k}^{\left(m\right)}-\frac{f\left(T_{j,k}^{\left(m\right)}\right)}{f^{\prime }\left(T_{j,k}^{\left(m\right)}\right)},\;f\left(T\right)=G_{corr}^{\left(j,k\right)}-t_{int}\cdot \bar{R}\cdot \bar{L}\left(T;\hat{\varepsilon }\right)$$

The value $\hat{T}$ obtained from the spectral-inversion stage is used as the initial estimate $T_{j,k}^{\left(0\right)}$. Because the emissivity function $\hat{\varepsilon }(\lambda )$ in $\bar{L}(T;\hat{\varepsilon })$ exhibits only weak temperature dependence through the weighting of the Planck function, $\hat{\varepsilon }(\lambda )$ is held fixed at each iteration using the coefficients retrieved by the GA, while temperature variation is represented only through the Planck function in $\bar{L}$. The convergence criterion is $\left|T_{j,k}^{\left(m+1\right)}-T_{j,k}^{\left(m\right)}\right|<10^{-4}\;K$. Under typical conditions, convergence is achieved within 3–5 iterations.

By applying the inversion to all valid pixels, a two-dimensional temperature distribution is reconstructed using the representative spectral emissivity function obtained from the spectral channel.

The emissivity function is obtained from a representative spectral sampling region and is then used in the radiometric equation for each valid image pixel. Thus, the method performs pixelwise temperature inversion with a transferred emissivity function; it does not independently determine emissivity at every pixel. This transfer is most appropriate when material composition and surface state are sufficiently similar across the region of interest. For spatially heterogeneous surfaces, multipoint or region-specific spectral measurements are required to reduce emissivity-transfer error.

### 2.3. Method Assumptions, Limitations, and Uncertainty Analysis

To define the applicability of the proposed method, this section systematically describes the principal assumptions and their physical basis, followed by an analysis of uncertainty propagation.

#### 2.3.1. Principal Assumptions and Applicability

##### Lambertian Approximation

The conversion between spectral exitance and radiance assumes approximately diffuse emission. In the present experiments, the observation geometry is fixed and close to the target-normal direction, which reduces but does not remove directional-emissivity effects. The approximation is more reasonable for rough or oxidized surfaces than for smooth, specular, or molten regions. In the laser-heated 316L case, melting and evolving surface morphology may increase the directional component of the radiation; temperatures reconstructed in such regions should therefore be interpreted with this limitation in mind. Dedicated directional-emissivity measurements would be needed to quantify the associated bias.

##### Temperature Dependence of Emissivity

The spectral stage retrieves an effective emissivity function for the measured state, and that function is subsequently held fixed when solving the image pixels. Real metallic emissivity can change with temperature, oxidation, roughness, and phase state. Consequently, transferring one emissivity function across a field containing a significant temperature or surface-state gradient can introduce systematic error. This assumption is relatively less restrictive for the steady tungsten–halogen source, but it is more important for the transient 316L experiment, where melting and oxidation may evolve during irradiation.

##### Spatial Representativeness of the Transferred Emissivity

The spectral emissivity is measured over a representative sampling region rather than independently at every image pixel. The same function can therefore be transferred only when the material composition and surface state within the image region are sufficiently similar to those in the spectral sampling region. Local oxidation, contamination, coating variation, melting, or phase heterogeneity can violate this condition. In the present setup, spatial correspondence is established by mounting the camera and fiber collimator on the same adjustment bracket and by visualizing the spectral sampling position through reverse illumination of the fiber channel. Residual field-of-view mismatch remains an uncertainty source. For heterogeneous targets, multipoint spectroscopy or region-specific emissivity functions should be used.

##### Validity of the Second-Order Emissivity Model

The second-order polynomial is appropriate only when emissivity varies smoothly over the 500–760 nm analysis band and does not contain strong narrowband structures. The measured spectra in the present steady-state and transient examples satisfy this smoothness condition sufficiently for the proof-of-concept analysis. The assumption should not be extended to gases, semiconductors near band edges, coated surfaces with interference features, or other materials with pronounced spectral structure without prior model comparison.

#### 2.3.2. Comparison with Existing Thermometry Methods

Table 2 provides a qualitative comparison with two-color and color-ratio pyrometry. Because temperature accuracy and system cost depend strongly on wavelength selection, detector configuration, calibration procedure, target emissivity, and operating conditions, unsupported universal percentages are not used as comparison criteria.

Compared with two-color and color-ratio approaches, the present method uses continuous spectral measurements to estimate a wavelength-dependent representative emissivity instead of requiring a gray-body or unity-emissivity-ratio assumption. This can reduce systematic bias when those assumptions are inadequate, provided that the emissivity model and spatial-transfer assumptions remain valid. The trade-off is the need for a spectrometer in addition to the imaging camera and for a numerical joint inversion. The resulting hardware architecture is compact, but the present study does not claim a universal cost advantage or a condition-independent temperature-accuracy advantage over commercial or two-color systems.

#### 2.3.3. Uncertainty Propagation Analysis

The uncertainty of the reconstructed temperature field can be organized into three coupled components: uncertainty in the spectral temperature–emissivity inversion, uncertainty introduced when the representative emissivity function is transferred to the imaging field, and uncertainty in the camera gray-level-to-radiance conversion. The following relations describe how these components propagate through the model; they are a sensitivity framework rather than a complete experiment-specific uncertainty budget.

Component I: spectral-inversion uncertainty. This component includes stochastic variation in the GA solution, spectrometer noise, and uncertainty in the absolute spectral-radiance calibration. Numerical simulations characterize the algorithmic sensitivity to imposed noise, but they do not replace a metrologically traceable uncertainty assessment of the experimental spectrometer channel.

Component II: emissivity-transfer uncertainty. Errors in the fitted emissivity coefficients propagate through the image radiometric equation to the pixel temperature. The local sensitivity can be estimated analytically as follows.

(24) $$\frac{\sigma_{T}^{\left(\varepsilon \right)}}{T}\approx \frac{{RMSE}_{\varepsilon }}{\bar{\varepsilon }}\cdot \frac{\bar{\lambda }T}{C_{2}}$$

Using the Wien approximation $\bar{L}\propto \bar{\varepsilon }\cdot exp(-C_{2}/(\bar{\lambda }T))$, and taking the logarithm of Equation (21) for a fixed corrected gray level $G_{corr}$, yields $ln\bar{\varepsilon }-C_{2}/(\bar{\lambda }T)=const$. Differentiation gives $d\bar{\varepsilon }/\bar{\varepsilon }+(C_{2}/(\bar{\lambda }T))\cdot (dT/T)=0$, and therefore $dT/T=-(\bar{\lambda }T/C_{2})\cdot (d\bar{\varepsilon }/\bar{\varepsilon })$. Thus, emissivity error is attenuated as it propagates to temperature. Physically, an overestimated emissivity requires a lower temperature to reproduce the same radiance. The attenuation factor is $\bar{\lambda }T/C_{2}$.

This sensitivity relation shows that an emissivity mismatch produces a systematic temperature bias whose magnitude depends on wavelength, temperature, and emissivity. In practice, the relevant emissivity error includes not only the fitting residual of the polynomial model but also spatial mismatch between the representative spectral sampling region and each image pixel. The latter contribution cannot be quantified from the present single-point spectral dataset.

Component III: image-channel uncertainty. Photon statistics, electronic readout noise, residual dark/background signal, radiometric-calibration error, and detector nonlinearity can all propagate through the gray-level-to-temperature mapping. Near a solved temperature, the sensitivity can be linearized as

(25) $$\sigma_{T}^{\left(CCD\right)}\approx \frac{\sigma_{G}}{t_{int}\cdot \bar{R}\cdot |\partial \bar{L}/\partial T|}$$

The corresponding temperature contribution should be evaluated using gray-level noise and calibration residuals measured under the actual exposure and signal level. Because the available dataset does not contain a complete dark/background sequence, saturation statistics, sensor-temperature study, and independent calibration uncertainty for each transient operating point, no single numerical value is assigned here as the experimental camera-noise uncertainty.

Combined uncertainty.

(26) $$u_{c}(T_{j,k})=\sqrt{{\left[\sigma_{T}^{\left(spec\right)}\right]}^{2}+{\left[\sigma_{T}^{\left(\varepsilon \right)}\right]}^{2}+{\left[\sigma_{T}^{\left(CCD\right)}\right]}^{2}}$$

If the component uncertainties are independently characterized, they can be combined using Equation (26), and the calculation can in principle be repeated pixel by pixel because signal level and local sensitivity vary across the image. For the present transient experiment, the required independent inputs are incomplete; therefore, the analytical framework is retained to identify the propagation paths, but a traceable experiment-specific combined uncertainty or two-dimensional uncertainty map is not reported.

## 3. Experimental Results and Analysis

### 3.1. Experimental Setup and Measurement Parameters

**Figure 2:**
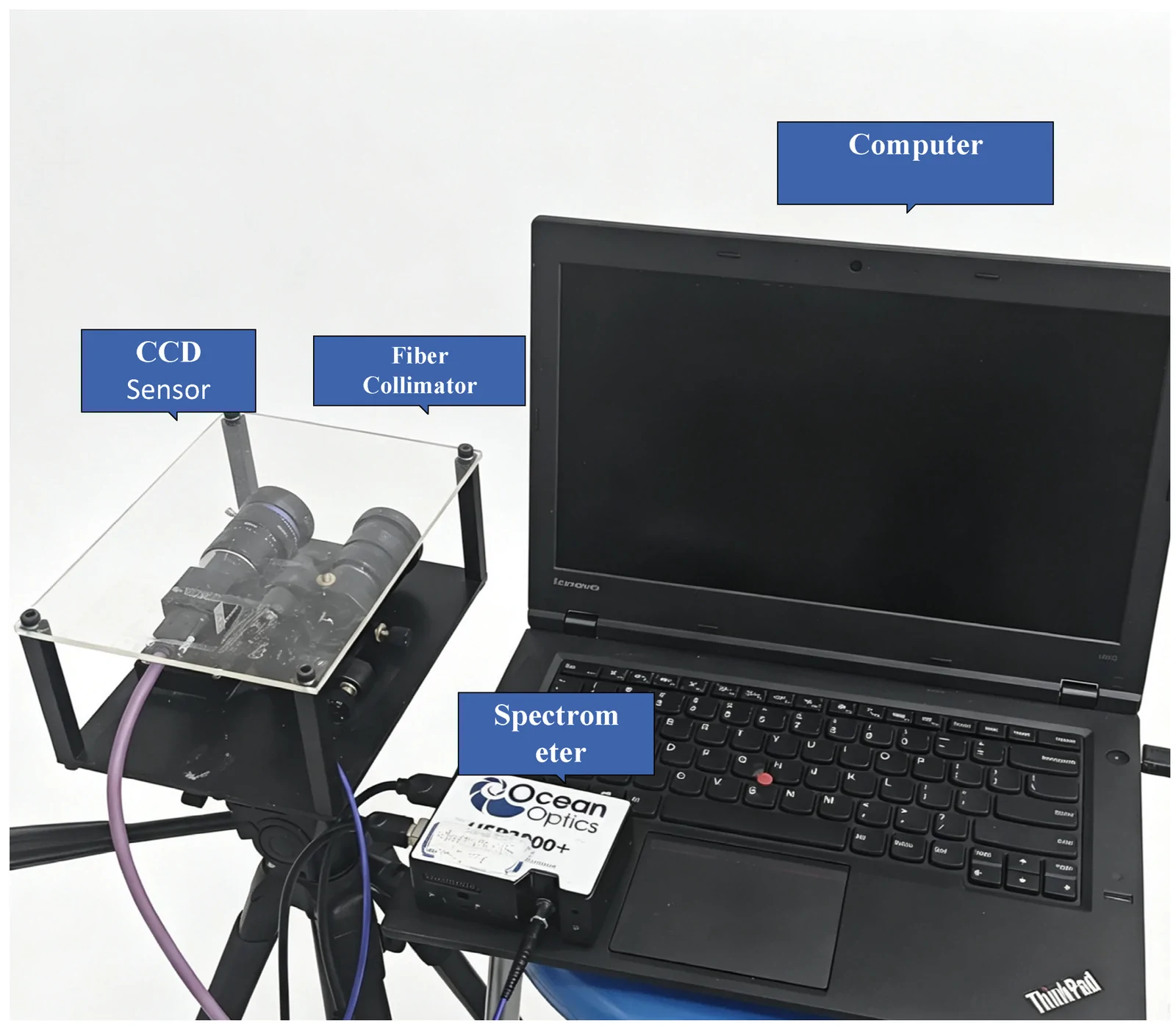
Experimental system and hardware layout for spectral–image fusion thermometry.

A dual-channel imaging–spectroscopy radiation-detection system was constructed, comprising an imaging unit, a fiber-collimation and coupling unit, and a spectral-detection unit. A photograph and layout of the system are shown in Figure 2. The camera and fiber collimator were mounted on the same precision adjustment bracket with approximately parallel optical axes to improve the spatial correspondence between the image field of view and the spectral sampling region. The complete system consisted of an industrial area-array camera (MV-CA003-21UM; HIKROBOT, Hangzhou, Zhejiang, China), a 100 mm C-mount telephoto imaging lens (ZLKC, Guangzhou, China), a 1000 nm short-pass optical filter (ZLKC, Guangzhou, China), a fiber collimator (FC-COL1825; Shenzhen Xinrui Optical Technology Co., Ltd., Shenzhen, China), an SMA905 energy-transmission fiber (Xinruiguang, Shenzhen, China), an Ocean Optics USB2000+ fiber-optic spectrometer (Dunedin, FL, USA), a synchronous-trigger module, and a host computer. The principal hardware specifications are listed in Table 3.

The imaging unit employed a HIKROBOT MV-CA003-21UM industrial area-array camera incorporating an onsemi PYTHON300 monochrome silicon global-shutter sensor. The sensor operates over a broader visible–near-infrared response range than the quantitative band used in this study. A 1000 nm short-pass optical filter (ZLKC, Guangzhou, China) was mounted in front of the 100 mm imaging lens (ZLKC, Guangzhou, China) to suppress longer-wavelength leakage. Although the detector and spectrometer cover broader nominal wavelength ranges, quantitative spectral-emissivity and temperature inversion in the present work is restricted to approximately 500–760 nm, where the calibrated response and signal quality are suitable for analysis.

To improve spatial sampling of distant high-temperature targets, a 100 mm telephoto lens (ZLKC, Guangzhou, China) was used. The camera exposure time was set to 0.04 ms in the steady-state tungsten–halogen-lamp experiment and to 0.08 ms in the laser-heating experiment on 316L stainless steel, thereby balancing signal intensity and temporal resolution.

The spectral-detection unit employed an Ocean Optics USB2000+ miniature fiber-optic spectrometer equipped with a Sony ILX511B linear-array detector. The detection range was 200–1100 nm, and the optical resolution under the present configuration was approximately 1.5 nm full width at half maximum (FWHM). The instrument used 16-bit analog-to-digital conversion, had a corrected linearity better than 99.8%, and supported integration times from 1 ms to more than 65 s. The spectrometer integration time was fixed at 1 ms, and an ESP32-based synchronous-trigger module coordinated acquisition with the camera, providing microsecond-level synchronization.

The radiation-coupling and transmission unit used an FC-COL1825 fiber collimator with an 18 mm clear aperture and a 25 mm focal length to collect radiation from the target. The signal was transmitted to the spectrometer through an SMA905 energy-transmission fiber (Xinruiguang, Shenzhen, China) with a 400 μm core and a 400–1100 nm operating range. SMA 905 connectors were used at both ends of the fiber.

During operation, the camera and fiber collimator remained fixed on the same adjustment bracket, and the two channels were synchronously acquired. Before measurement, a 520 nm laser was coupled backward through the fiber so that the fiber-collimator channel projected a visible spot onto the target plane. The collimator position and pointing were adjusted until this reverse-projected spectral sampling spot lay within the camera region of interest used for subsequent temperature inversion. The alignment image was retained to define the spatial computation window. This procedure establishes a practical correspondence between the spectral sampling region and the image, although the present records do not support a numerical residual-registration error or sampling-spot diameter.

### 3.2. Calibration of the CCD Thermometry Model

**Figure 3:**
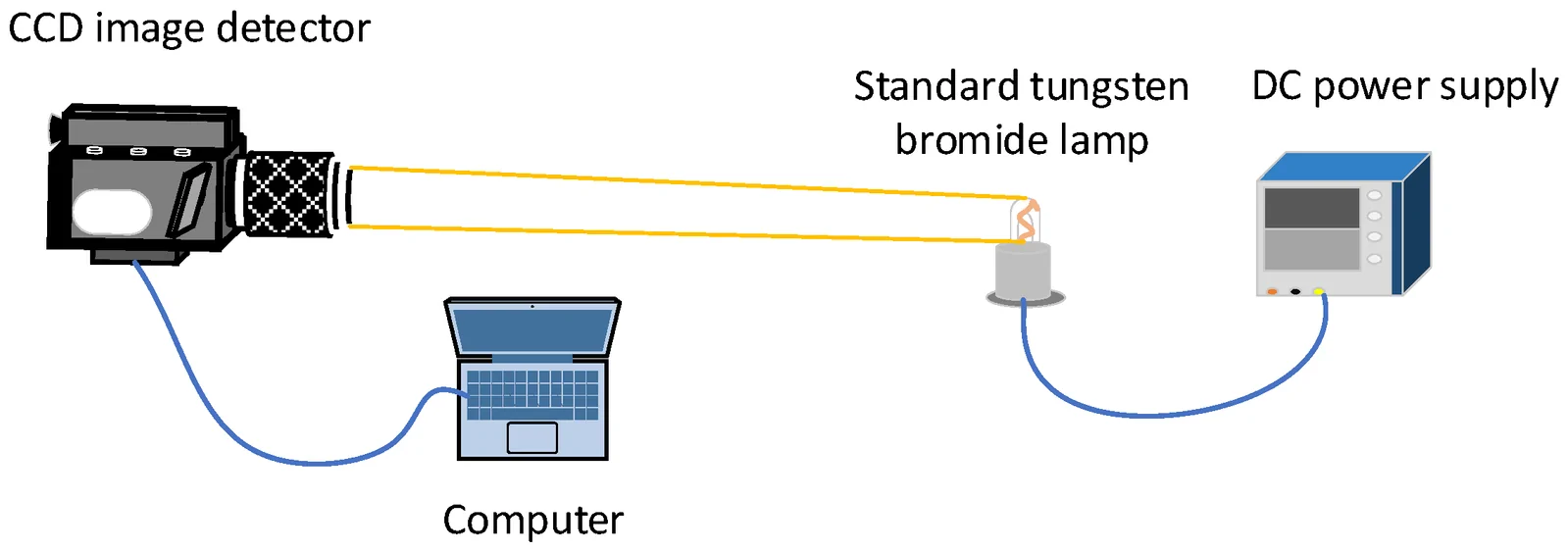
Schematic of the calibration setup.

Conventional radiometric calibration is commonly performed at one or more fixed exposure conditions. In this study, integration time is introduced explicitly into an empirical linear-response model so that measurements obtained at different calibrated exposure settings can be described by one parameterized relationship. Calibration measurements were performed with a metrology-calibrated tungsten–halogen radiation source over nominal color-temperature settings of 2200–2800 K. The color-temperature operating points follow the calibration certificate of the SL3186 standard color-temperature tungsten lamp (YouLai Technology, Hangzhou, China; certificate No. UL25100234) in operating state before spectral and image data acquisition. The 2200–2800 K values therefore denote metrologically calibrated CCT operating points, and the certificate provides color-temperature calibration rather than wavelength-by-wavelength absolute spectral radiance calibration; the lamp thus serves as a stable, repeatable, metrologically traceable radiation source rather than as an absolute spectral radiance standard. The nominal color temperature is used only as an operating-state label and is not treated as the filament thermodynamic temperature or as evidence that the lamp is an ideal blackbody over the full VIS–NIR range. Quantitative spectral processing uses calibrated spectral-radiance information over approximately 500–760 nm. A schematic of the calibration hardware is presented in Figure 3. The calibration model is applied only within the experimentally established camera linear-response and radiance ranges, which was calibrated in accordance with JJF 1976–2022, “Calibration Specification for Average Color Temperature Standard Lamps,” over 1600–3000 K. The certificate specifies lamp currents of 2.673, 2.857, 3.045, 3.239, 3.435, 3.635, and 3.843 A corresponding to correlated color-temperature (CCT) operating points of 2200, 2300, 2400, 2500, 2600, 2700, and 2800 K, with an expanded uncertainty of U = 18 K (k = 2, approximately 95% coverage probability). The operating points were set according to the certificate currents, and a stabilization time of no less than 8 min was maintained after each change.

**Figure 4:**
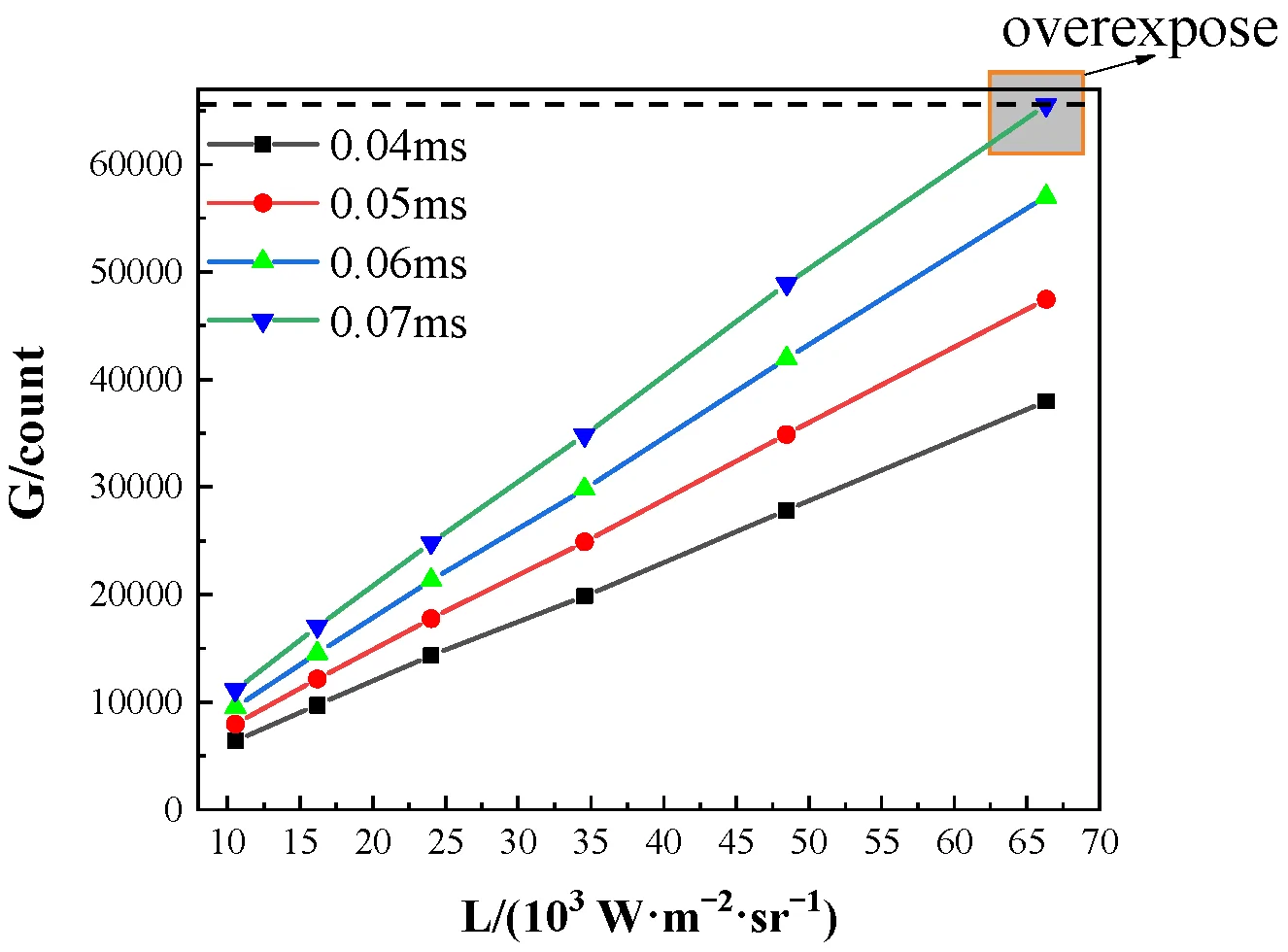
Relationship between radiance and output gray level at different integration times.

To establish the usable linear-response domain, gray-level images were acquired at several source levels and integration times. Only data that remained sufficiently above the noise floor and below saturation were included in the linear fit. Figure 4 shows the measured relationship between reference radiance and gray level for the tested integration times. The observed linearity supports use of the three-parameter model within this tested domain; it does not imply valid extrapolation to arbitrarily short, long, underexposed, or saturated exposures.

The multi-integration-time calibration curves therefore provide experimental support for interpolation within the measured linear-response region. When the camera is operated outside this region, low-signal noise or saturation-related nonlinearity invalidates the assumed gray-level–radiance–integration-time relationship.

(27) G(t,T)=14.1326 t L(T)+12552.4506t+7.718

**Figure 5:**
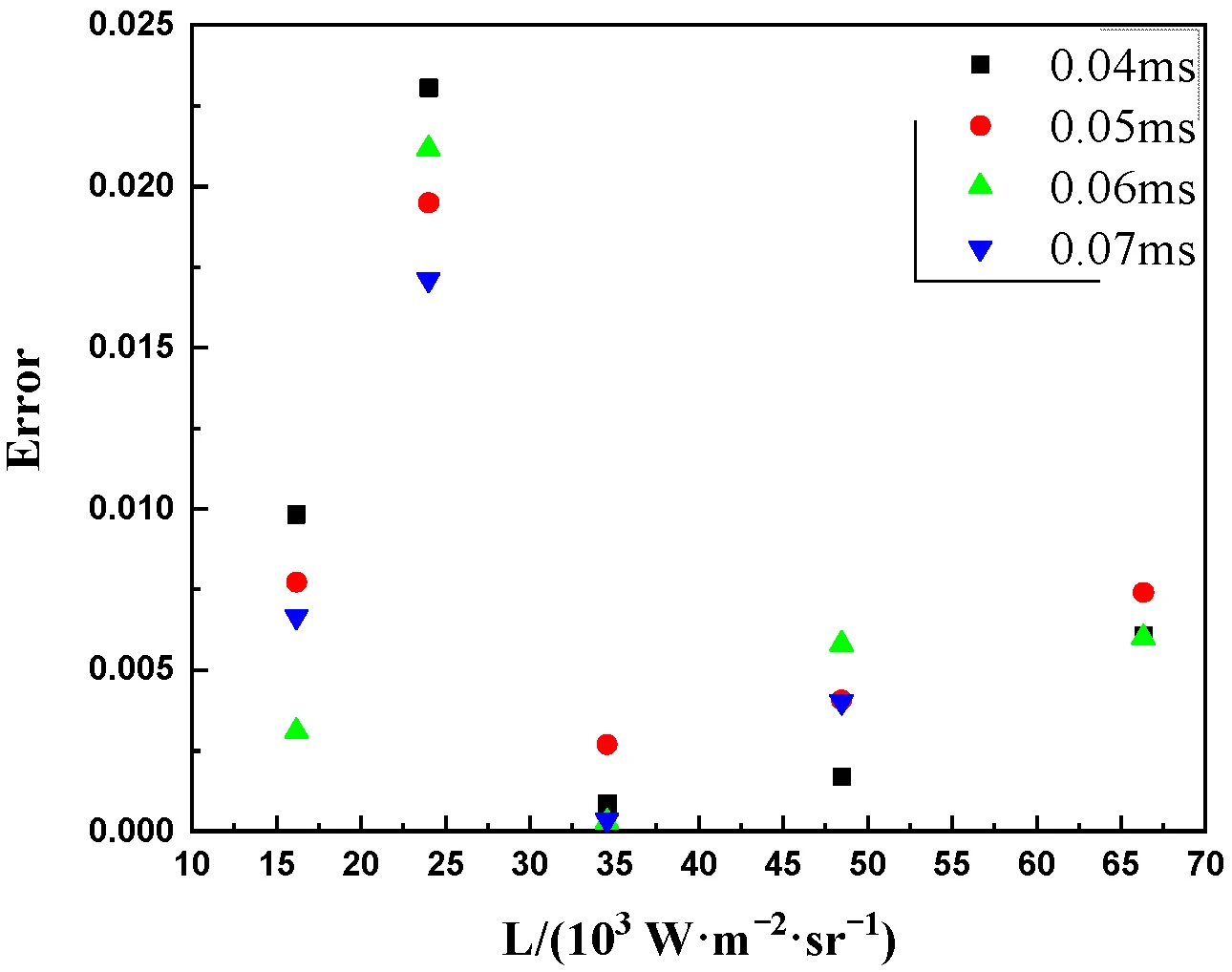
Error of the radiometric calibration model at different integration times.

The fitted calibration relationship was used to reconstruct gray-level responses at the integration times contained in the calibration dataset, and the residuals relative to the corresponding reference-radiance data are summarized in Figure 5. These residuals characterize the internal fit of the available calibration data; because an independent integration time was not withheld from parameter fitting, they should not be interpreted as a leave-one-exposure-out validation of the model.

A stronger future validation will reserve one or more integration times exclusively for prediction and will report gray-level, radiance, and equivalent-temperature prediction errors. The archived calibration records used for the present manuscript do not contain the complete per-exposure arrays needed to perform that independent test without introducing reconstructed or assumed data.

### 3.3. Steady-State Temperature-Field Measurement with Spectral Emissivity Transfer

#### 3.3.1. Construction and Spectral Calibration of the Steady-State Radiation Field

To examine the thermometric behavior of the proposed framework under a stable radiation condition, four tungsten–halogen-lamp operating states with nominal color temperatures of 2200, 2400, 2600, and 2800 K were used. These operating states correspond to certificate-specified current settings of the SL3186 standard color-temperature lamp (YouLai Technology, Hangzhou, China; Section 3.2); each operating point was stabilized for at least 8 min before data acquisition. The camera and fiber-optic spectrometer observed the aligned target region synchronously. The spectrometer integration time was 1 ms and the camera exposure time was 0.04 ms. The nominal color temperature describes the spectral-equivalence operating state of the lamp and is not taken as the thermodynamic temperature of the filament. The absolute spectral-radiance calibration and the color-temperature label are therefore treated as distinct quantities.

**Figure 6:**
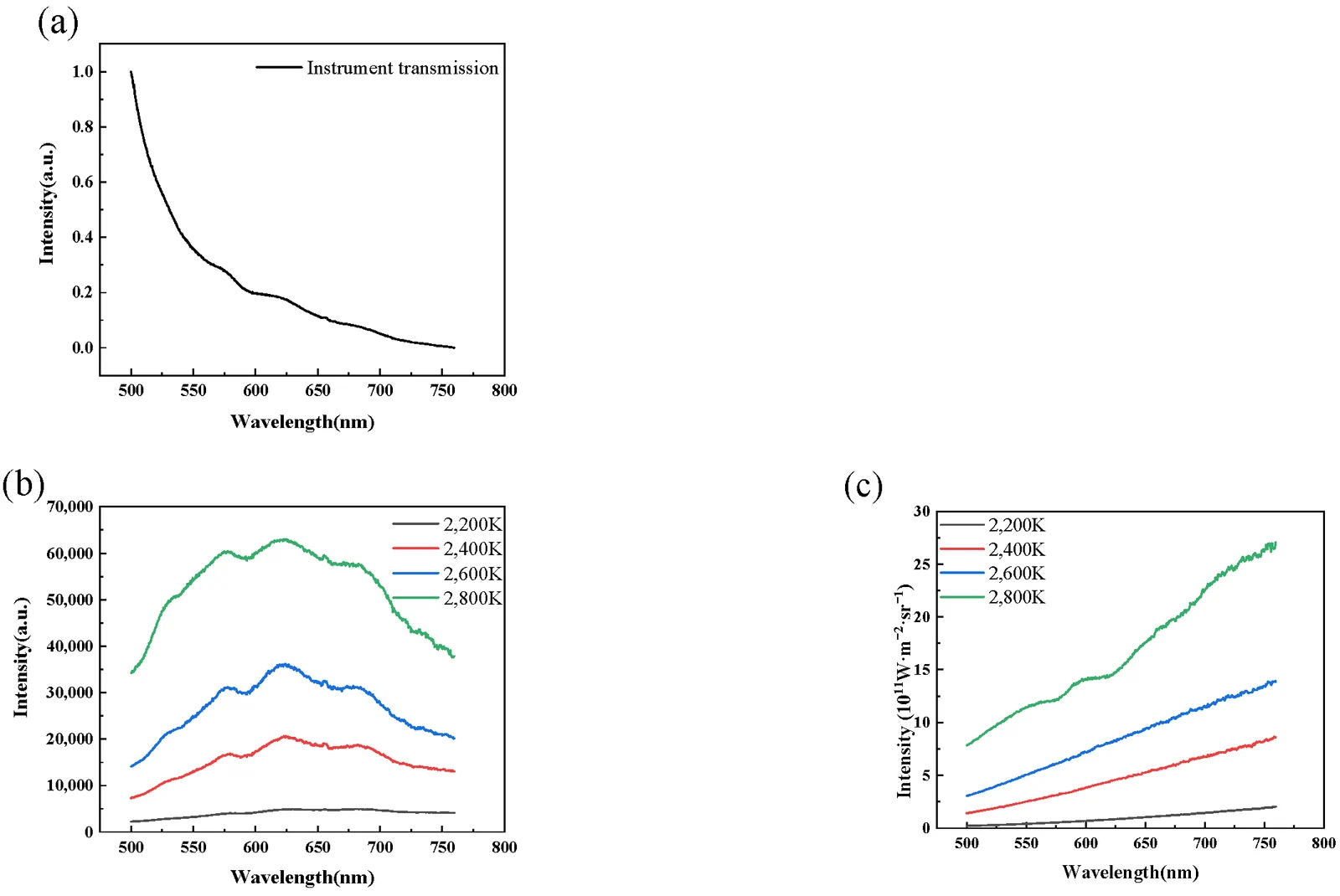
Spectral-calibration results for the steady-state radiation field of the tungsten–halogen lamps: (**a**) normalized system spectral-response function; (**b**) relative spectral-radiation intensity at different nominal color temperatures; (**c**) absolute spectral-radiation intensity after radiometric calibration.

Relative spectra were acquired over 500–760 nm and converted to calibrated spectral-radiance data using the system response, as shown in Figure 6. The spectra increased with source operating level and remained continuous and smooth over the analysis band, providing the spectral conditions required for the low-order emissivity model. The uncertainty of the absolute spectral-radiance reference is a separate metrological contribution and should be included in a complete uncertainty budget; it cannot be inferred from the nominal lamp color temperature alone. The color-temperature certificate (U = 18 K, k = 2) characterizes the metrological traceability of the source operating state, whereas the absolute spectral-radiance uncertainty requires independent radiance calibration and is not provided by the color-temperature certificate.

#### 3.3.2. Emissivity-Model Selection and Spectral Temperature Inversion

**Figure 7:**
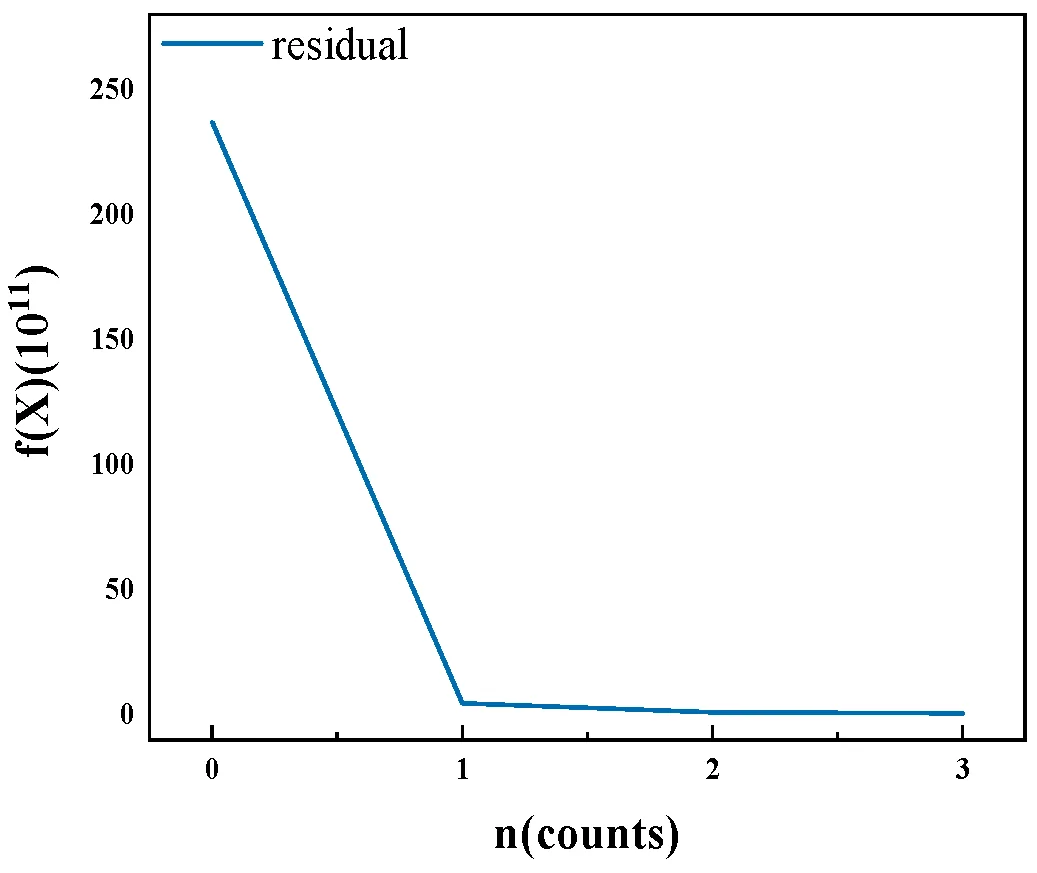
Residual comparison for emissivity polynomial models of different orders.

First-, second-, and third-order polynomial emissivity models were compared over 500–760 nm. The unnormalized residual sums shown in Figure 7 decrease substantially from first to second order, whereas the additional decrease from second to third order is limited. Because the residuals are not normalized by signal magnitude, Figure 7 is used only as qualitative support for the order-selection trend and not as a cross-condition normalized performance metric. Together with the AIC-based complexity argument in Section 2.1.2, this supports use of the second-order model for the present proof-of-concept analysis. A material-specific AIC/BIC and temperature-sensitivity reanalysis for tungsten and 316L remains future work.

**Figure 8:**
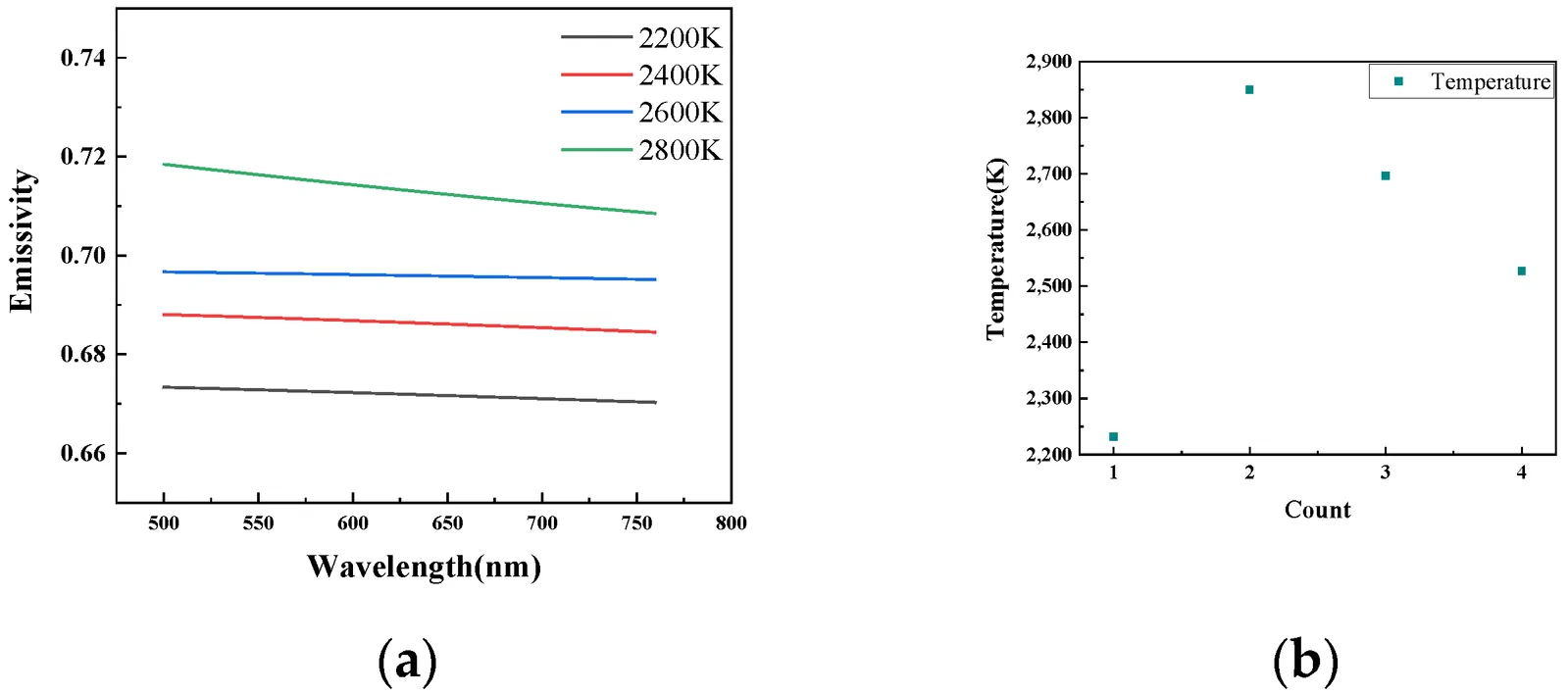
Steady-state spectral-inversion results for the tungsten–halogen lamps: (**a**) spectral emissivity over 500–760 nm; (**b**) retrieved temperature under each operating condition.

The radiometrically calibrated absolute spectral-radiation intensities were input into the genetic algorithm described in Section 2.1. Temperature T and the second-order emissivity coefficients (a_0_, a_1_, and a_2_) were treated as unknown parameters, and the physical constraint 0 < ε(λ) < 1 was imposed to jointly retrieve temperature and spectral emissivity. Figure 8a shows that the emissivity curves for all four operating conditions vary continuously and slowly over 500–760 nm and decrease slightly with wavelength. The corresponding temperature-inversion results are shown in Figure 8b. The retrieved temperatures were 2231.54, 2526.79, 2696.78, and 2849.69 K, respectively, increasing monotonically with nominal color temperature. This result indicates that the model can stably characterize temperature variations at different steady-state radiation levels.

#### 3.3.3. Temperature-Inversion Results and Internal Consistency Check

**Figure 9:**
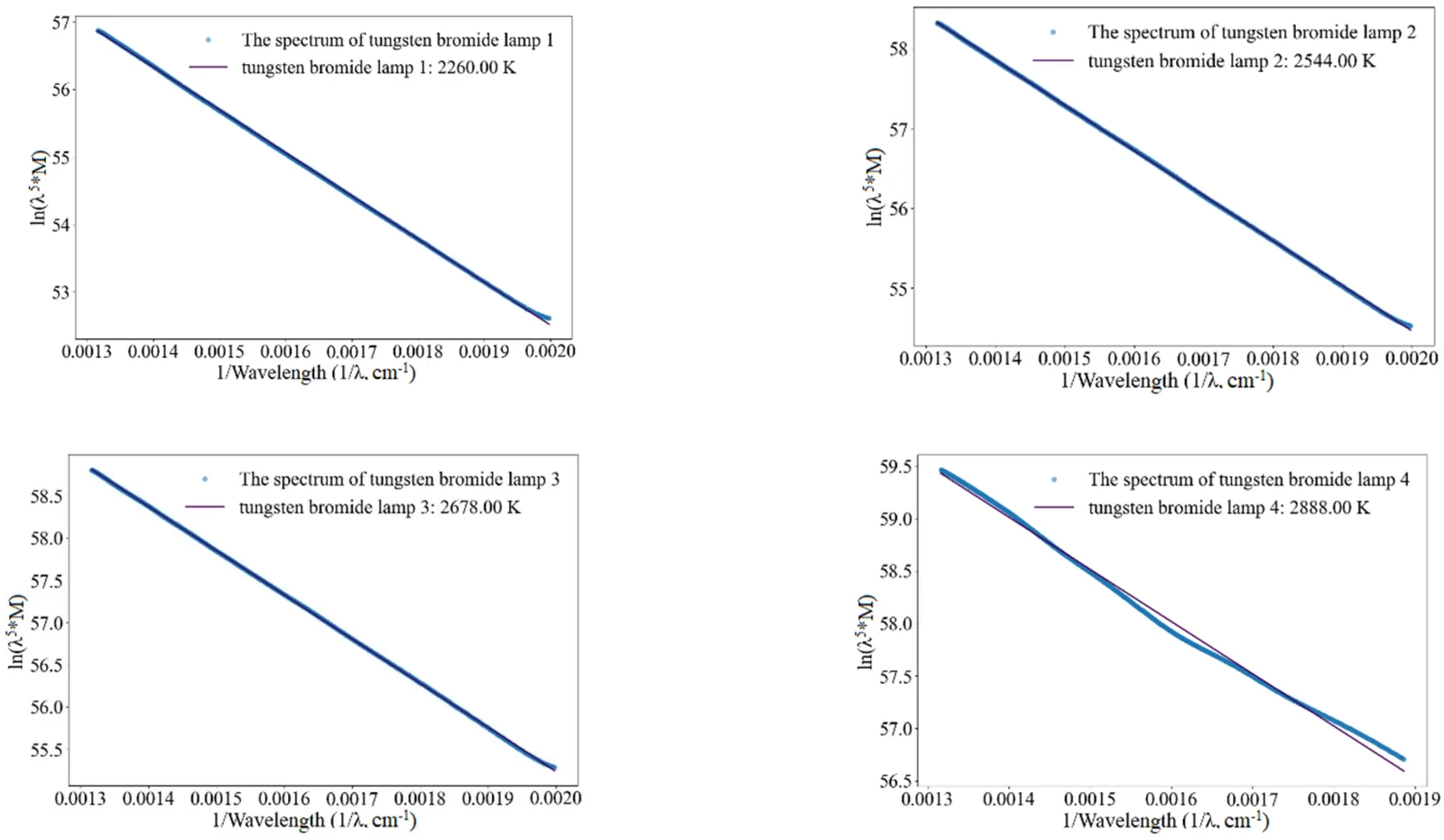
Temperature fitting based on the Wien-approximation relationship.

As an internal consistency check, the four calibrated spectra were also processed using the wavelength dependence implied by the Wien approximation, yielding the corresponding spectral-shape temperature estimates T_W shown in Figure 9. The joint-inversion temperatures T, nominal lamp color temperatures T_c, and Wien-based estimates T_W are summarized in Table 4. Because the proposed inversion and the Wien estimate both originate from the same measured radiation spectra, this comparison does not constitute an independent traceable validation of absolute thermodynamic temperature.

Comparison of the three temperature quantities is therefore interpreted as follows: T_c is the nominal lamp color-temperature label, whose calibrated operating points carry an expanded uncertainty of U = 18 K (k = 2) according to the SL3186 calibration certificate (Section 3.2), T_W is a temperature estimate derived from spectral shape under the Wien approximation, and T is the temperature retrieved by the joint radiance–emissivity model under the assumptions stated in Section 2.3. This color-temperature uncertainty characterizes the metrological traceability of the source operating state and should not be conflated with the uncertainty of the absolute spectral radiance in the 500–760 nm analysis band. Their agreement can demonstrate internal consistency of the spectral processing but cannot replace validation with an independently calibrated radiation thermometer or traceable blackbody source.

The joint-inversion temperatures differed from the Wien-based estimates by 17.21–38.31 K, with a maximum relative deviation of 1.33% between the two spectral estimates. This indicates that the two processing routes produce similar trends and values for the same measured spectra. The comparison should not be interpreted as a 1.33% absolute thermometric accuracy because no independent traceable reference temperature was measured in this experiment. The differences between T and the nominal color-temperature labels likewise do not prove that either value is the filament thermodynamic temperature. The present steady-state experiment therefore supports feasibility and internal spectral consistency, while absolute accuracy requires a separate calibrated radiometric reference.

#### 3.3.4. Reconstruction of the Two-Dimensional Steady-State Temperature Field

**Figure 10:**
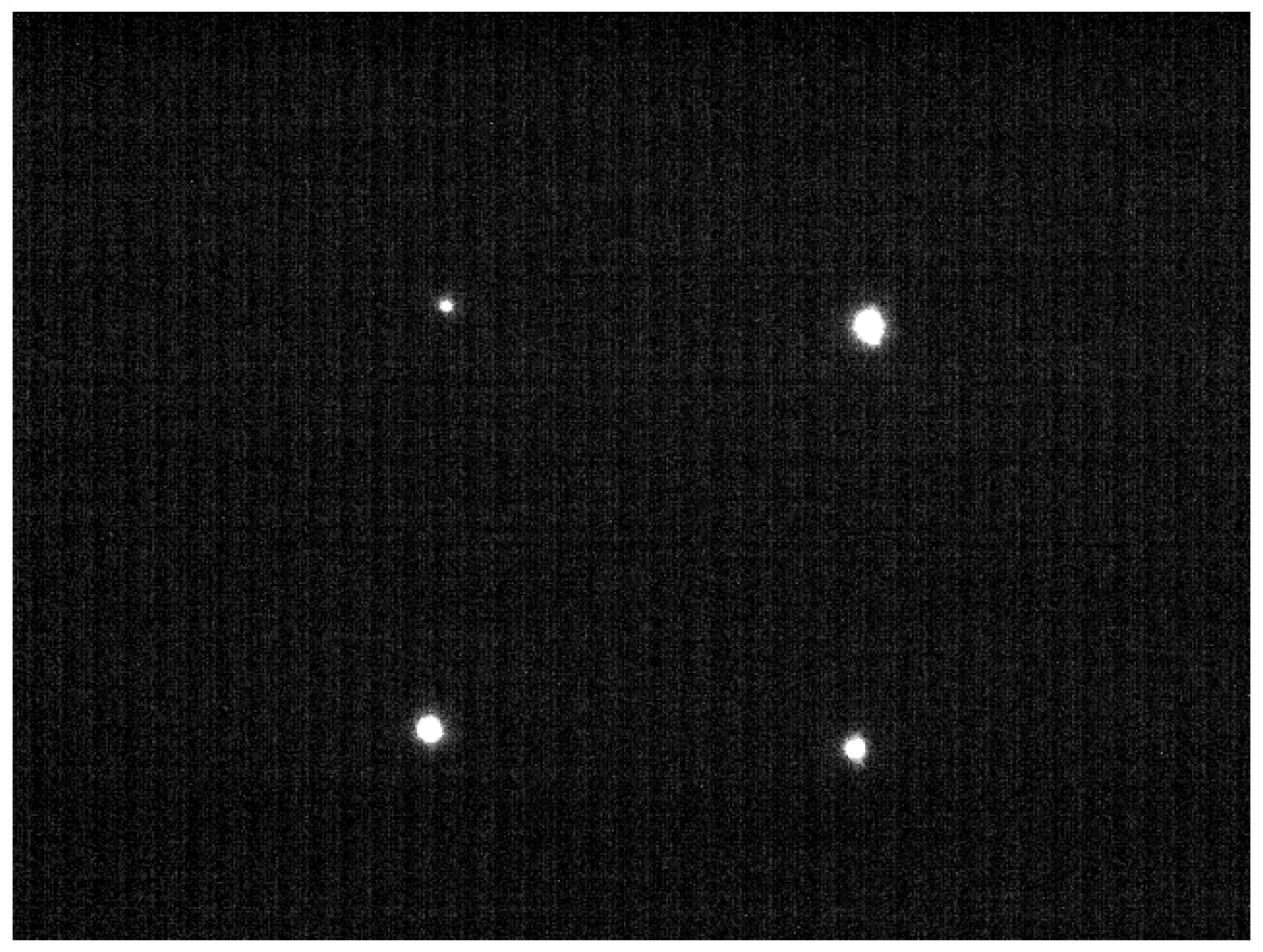
Steady-state gray-level images of the tungsten–halogen lamps at different nominal color temperatures.

After the representative spectral inversion, the retrieved emissivity function was transferred to the two-dimensional imaging stage. The camera exposure time was 0.04 ms, and the acquired gray-level images are shown in Figure 10. The calibration model converts valid pixel gray levels to equivalent radiance after accounting for the exposure-dependent background term and the electronic readout baseline.

**Figure 11:**
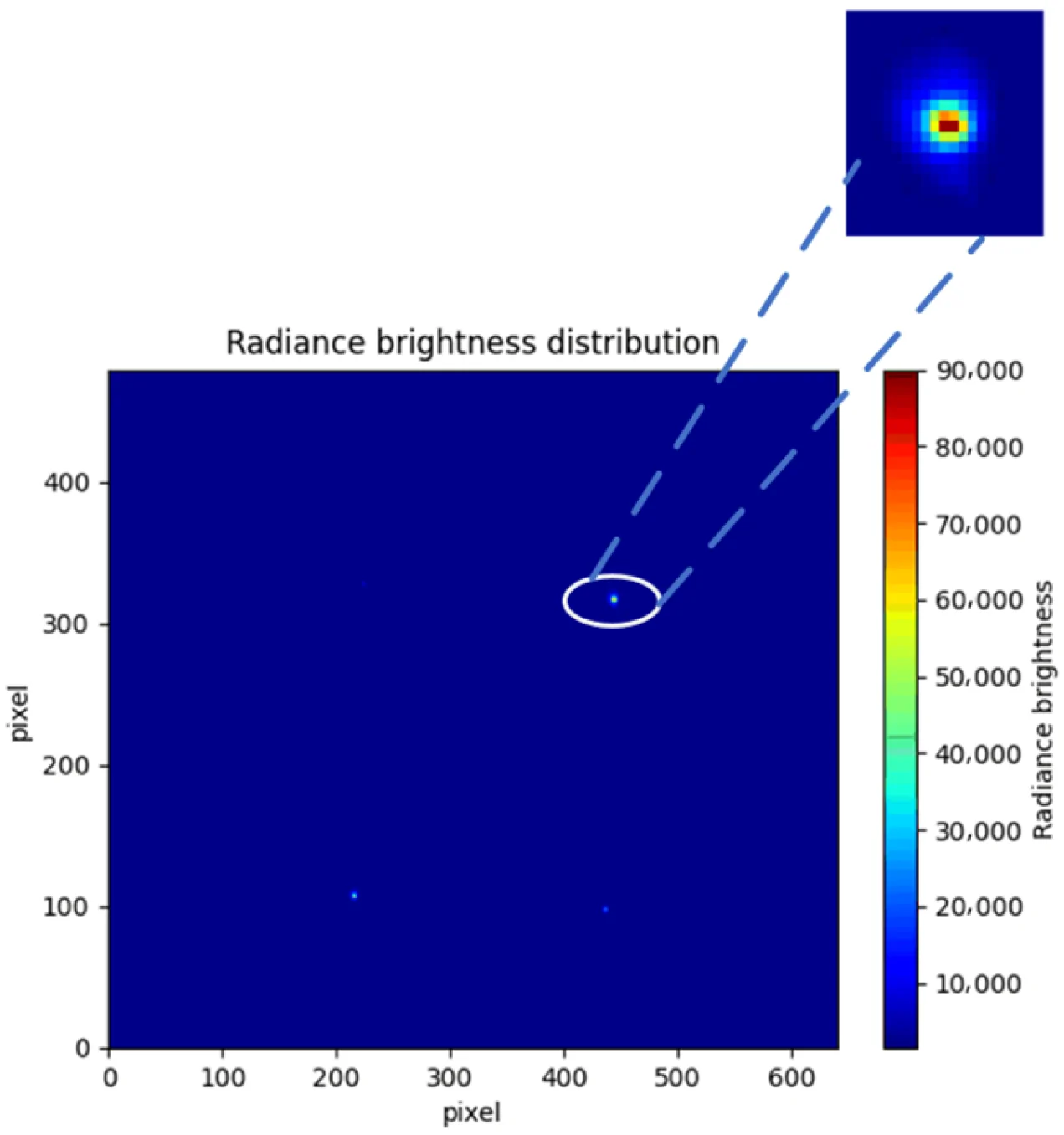
Steady-state radiance distribution of the tungsten–halogen lamps.

The two-dimensional radiance distribution retrieved from the CCD gray-level images is shown in Figure 11. The high-radiance regions coincide with the positions of the four tungsten–halogen lamps in the original image, indicating that gray-level correction and radiometric calibration preserve the spatial structure of the targets and provide reliable input for pixelwise temperature determination.

**Figure 12:**
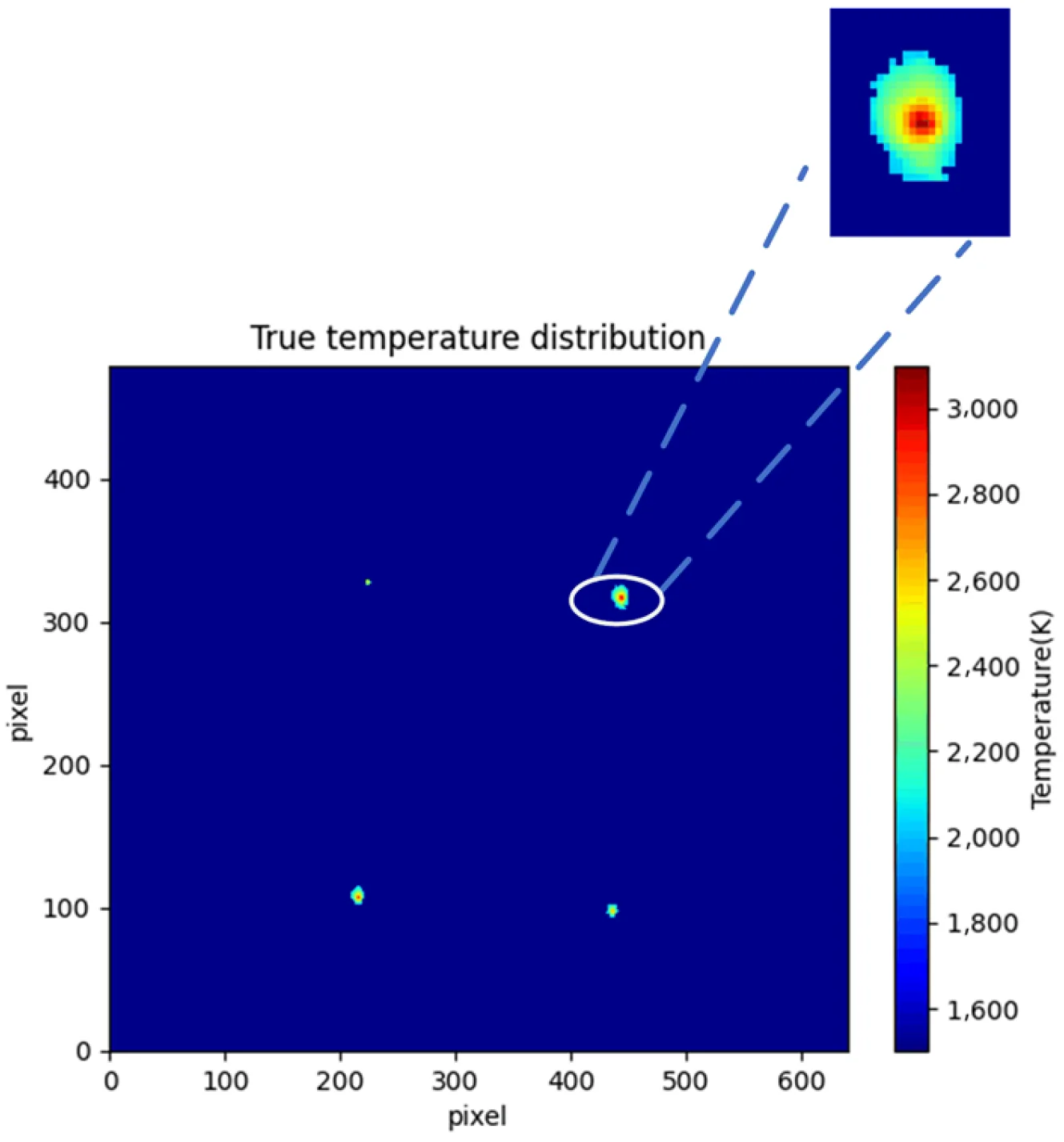
Two-dimensional steady-state temperature field of the tungsten–halogen lamps after spectral-emissivity correction.

The continuous second-order emissivity function obtained from the representative spectral region was then introduced into the pixel radiometric equation, and the temperature at each valid image pixel was solved by Newton–Raphson iteration. The same fitted emissivity function was used throughout the selected image region rather than independently re-estimated at each pixel. The reconstructed two-dimensional steady-state temperature field is shown in Figure 12; interpretation of this field therefore depends on the spatial-representativeness assumption discussed in Section 2.3.1.

Figure 10, Figure 11 and Figure 12 show consistent spatial correspondence among the gray-level images, radiance distributions, and reconstructed temperature fields. For the stable tungsten–halogen sources, using one representative emissivity function over each aligned radiation region is a reasonable approximation because the material and operating state are comparatively uniform. This case therefore provides a controlled demonstration of the point-to-field transfer procedure, rather than a validation of spatially resolved emissivity at every pixel.

### 3.4. Transient Temperature Inversion of Laser-Heated 316L Stainless Steel

**Figure 13:**
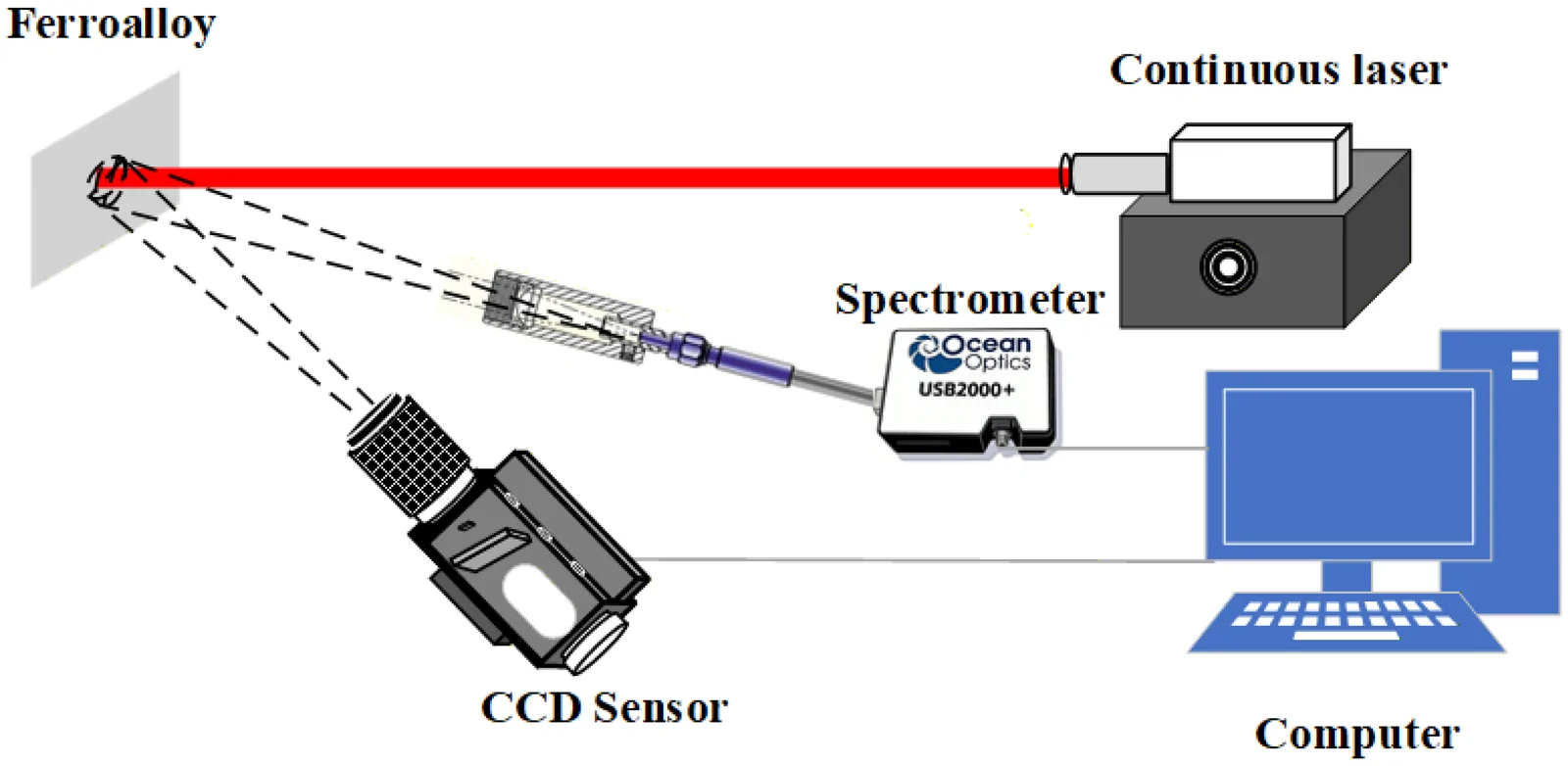
Schematic of the laser-heating experimental setup.

During the transient experiment, the laser beam was incident normally on the 316L stainless-steel surface. The spectrometer and imaging camera observed the heated region from separate but aligned channels and were synchronously triggered. The spectrometer collected radiation through the optical coupling path while the camera recorded the two-dimensional gray-level evolution. A schematic of the experimental arrangement is shown in Figure 13. Key thermophysical parameters for the test material are summarized in Table 5. The spatial correspondence between the two channels was established using the reverse-illumination procedure described in Section 3.1; incomplete overlap remains a potential source of emissivity-transfer error.

**Figure 14:**
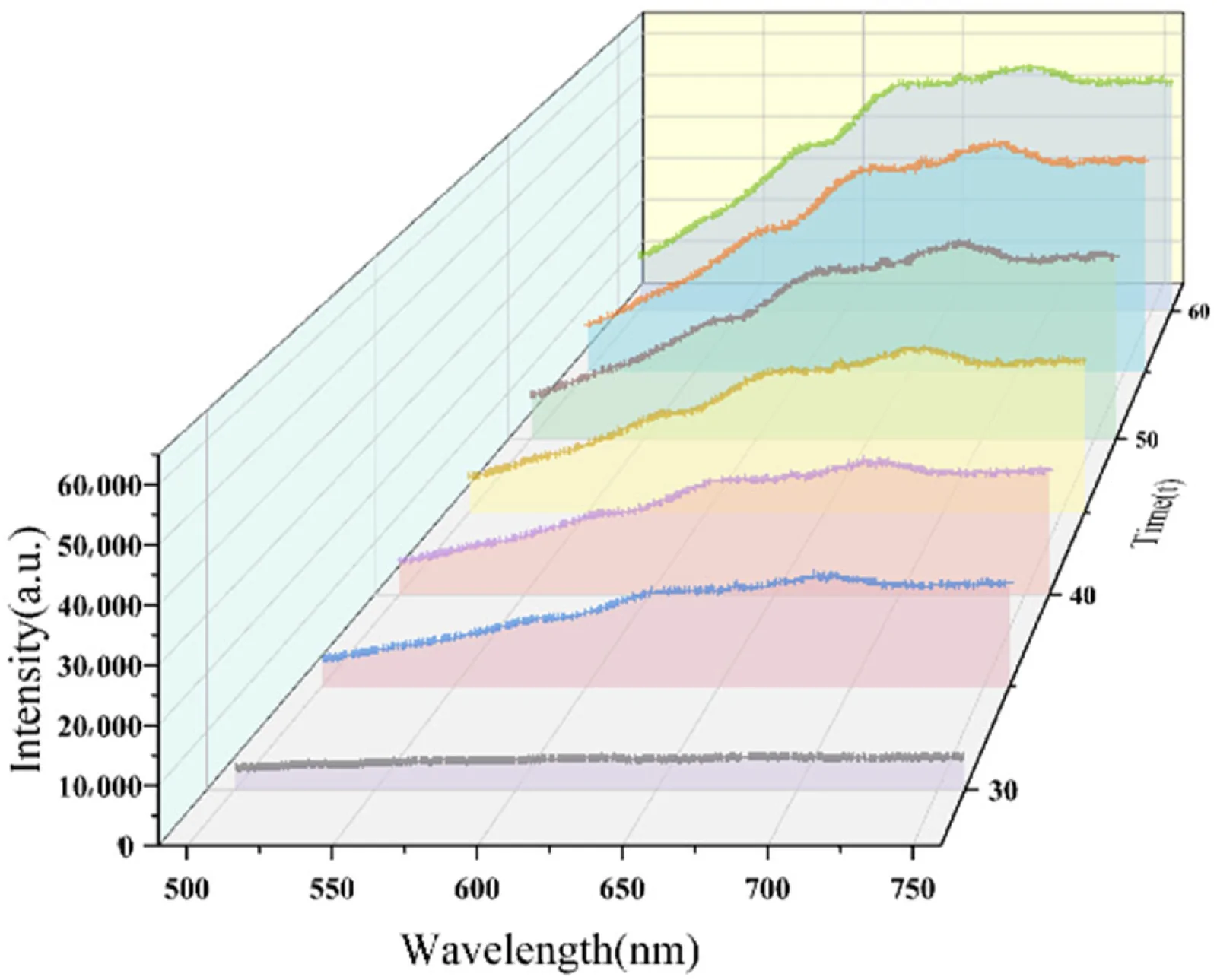
Relative spectral-radiation intensity of the 316L stainless-steel surface at different times.

The USB2000+ spectrometer continuously acquired the relative spectral-radiation intensity from the 316L stainless-steel surface during laser irradiation, yielding the time-dependent spectral-evolution curves shown in Figure 14.

#### 3.4.1. Spectral-Radiation Evolution and Temperature Inversion

As shown in Figure 14, within the visible range of approximately 500–760 nm, the spectrum at each time increased with wavelength and shifted overall toward higher intensity as the laser-interaction time increased. This band lies on the short-wavelength side of the high-temperature thermal-radiation spectrum, where increasing temperature produces a pronounced nonlinear increase in visible-band radiation intensity. Compared with the short-wavelength end, the separations among the curves became larger beyond 650 nm, indicating that the longer-wavelength region was more sensitive to temperature changes in this experiment and provided higher-SNR information for subsequent multiwavelength temperature inversion.

In terms of temporal evolution, the spectral intensity increased relatively slowly during the early stage, whereas the curves rose more markedly near 50–60 s, indicating substantial heat accumulation at the target surface. The spectra remained generally continuous and smooth at all times, with no pronounced isolated narrow peaks, showing that the dominant signal within the analysis band was continuous thermal radiation. This characteristic facilitates absolute radiometric calibration using the system spectral-response function and supports joint retrieval of temperature and emissivity with a continuous emissivity model.

**Figure 15:**
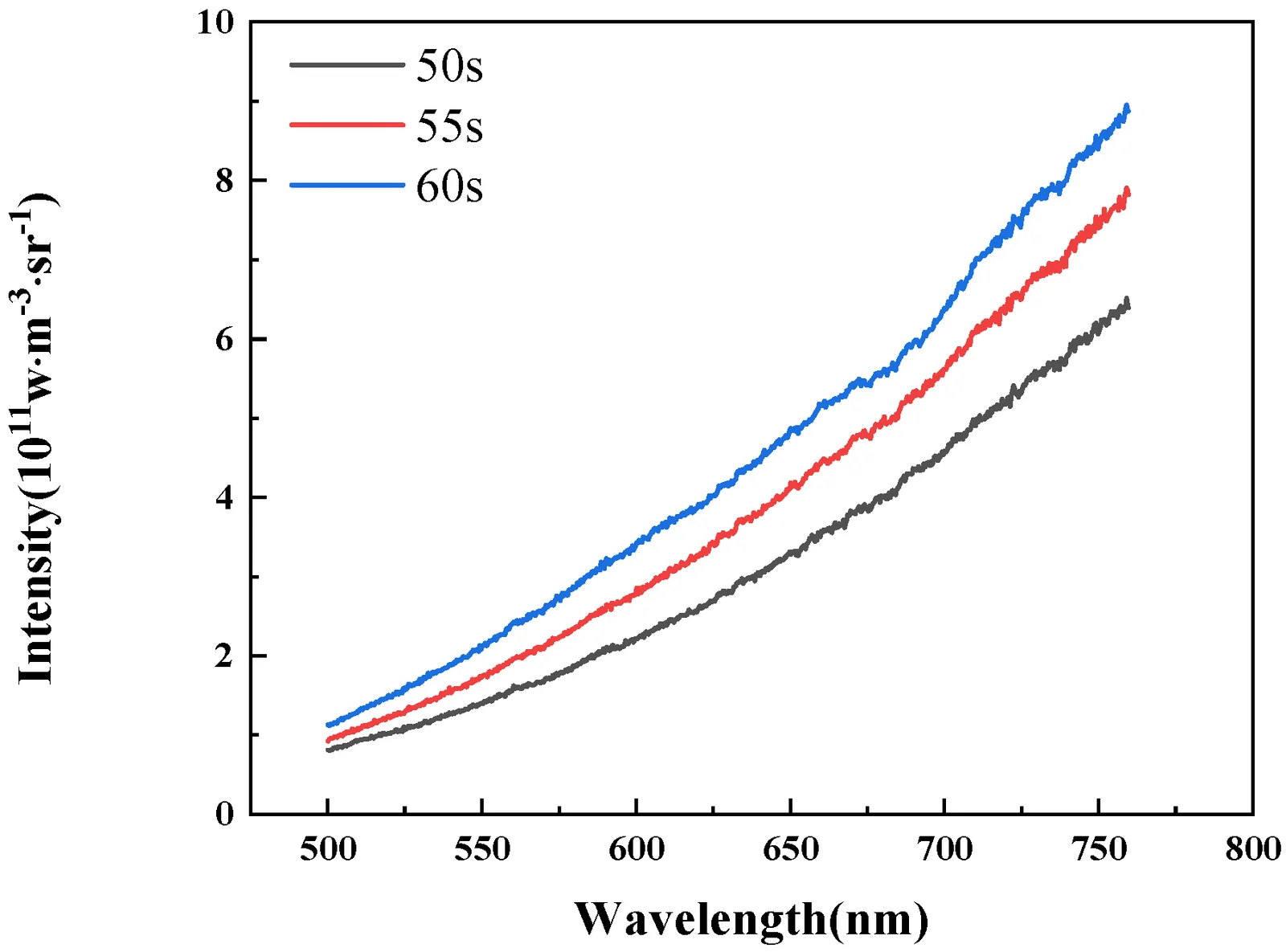
Absolute spectral-radiation intensity of the laser-heated 316L stainless-steel surface.

For quantitative analysis of the temperature variation, spectra at three representative times, 50, 55, and 60 s, were selected. The relative radiation intensities were converted into absolute spectral-radiation intensities using the system spectral-response function, as shown in Figure 15.

After absolute radiometric calibration, the three curves retained the order 60 s > 55 s > 50 s throughout the analysis band and exhibited no pronounced intersections, indicating that the spectral-response correction did not alter the temporal evolution present in the raw data. All three curves rose increasingly rapidly with wavelength, and the 60 s curve showed the largest increase at the long-wavelength end, suggesting a more rapid rise in surface temperature during 55–60 s. The stable ordering of the absolute radiation intensities also provides a basis for the monotonicity of the subsequent temperature-inversion results.

**Figure 16:**
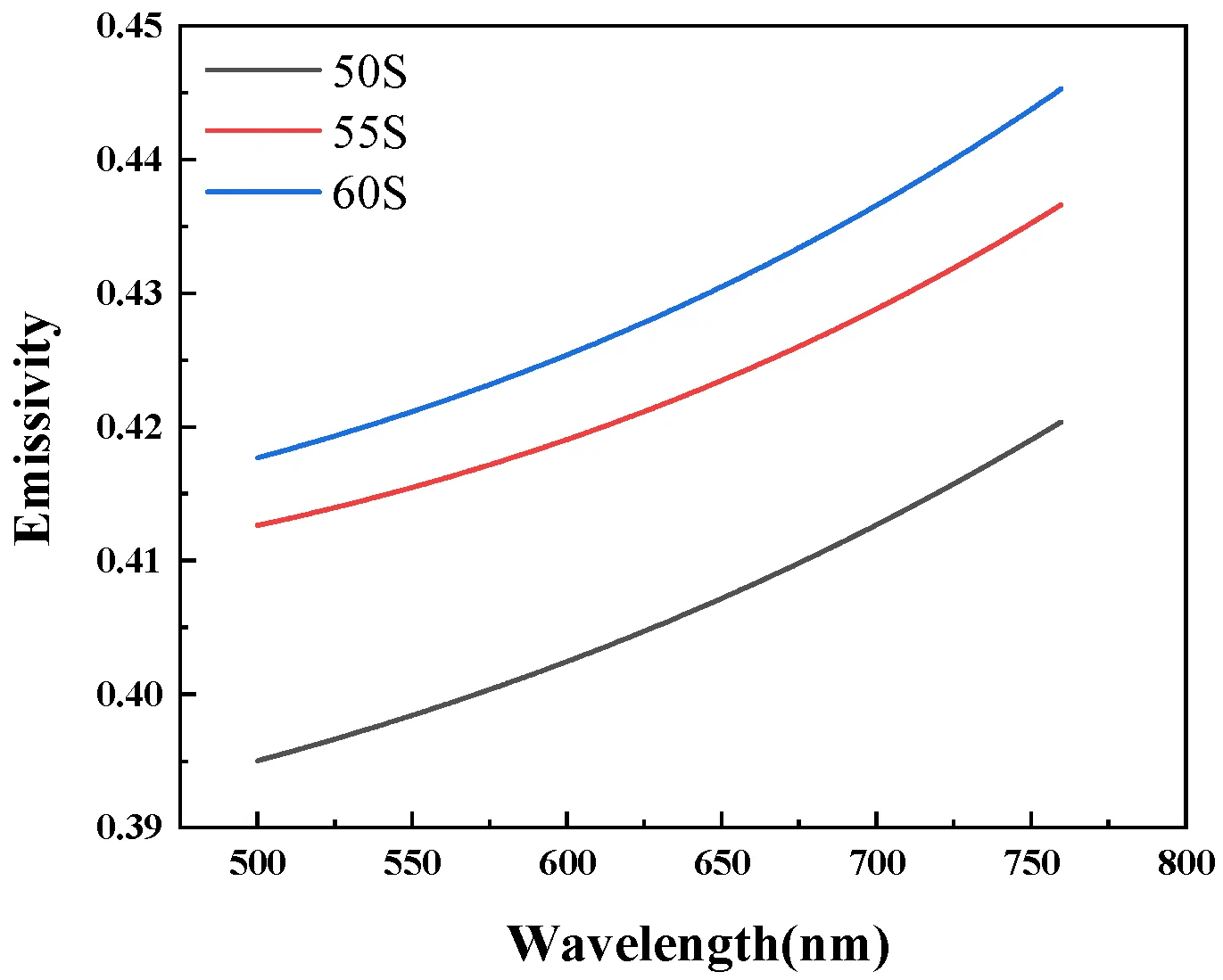
Spectral emissivity of the laser-heated 316L stainless-steel surface.

The absolute spectral-radiation intensities at different wavelengths were treated as known quantities. A polynomial was used to describe the wavelength-dependent emissivity of the 316L stainless-steel surface, and temperature and emissivity were solved simultaneously by iteration. The retrieved temperatures at 50, 55, and 60 s were 2311.92, 2336.05, and 2398.54 K, respectively. The temperature-change characteristics are listed in Table 6, and the corresponding spectral-emissivity curves are shown in Figure 16.

As shown in Table 6, the temperature increased by 24.13 K from 50 to 55 s, corresponding to a mean heating rate of 4.83 K·s^−1^. From 55 to 60 s, the temperature increased by 62.49 K and the mean heating rate rose to 12.50 K·s^−1^, approximately 2.59 times that of the preceding interval. Over 50–60 s, the cumulative temperature rise was 86.62 K, corresponding to an increase of 3.75% relative to 50 s and an overall mean heating rate of 8.66 K·s^−1^. Thus, the target surface did not heat at a constant rate but exhibited a pronounced acceleration during the later stage of laser heating.

The retrieved spectral-field-of-view temperatures at 50, 55, and 60 s exceed the tabulated 316L melting point of 1679 K. Because laser heating is strongly spatially nonuniform, this does not imply that the entire imaged region was fully molten. A more appropriate interpretation is that the observed region could contain a high-temperature molten or melt-pool core surrounded by cooler material. The spectrometer reports a radiation-weighted effective temperature over its sampling region, and the high-temperature portion can contribute disproportionately to the measured radiance. Melting, oxidation, evolving roughness, and melt-pool flow can all change directional radiative properties and spatial emissivity, so the use of one representative emissivity function is an approximation in this transient case.

Accordingly, the values 2311.92–2398.54 K should be interpreted as effective temperatures associated with the spectrometer sampling field under the adopted radiation model, not as proof that every point in that region has the same local thermodynamic temperature. The imaging stage provides a spatial temperature distribution using the representative emissivity function, but the accuracy near melt-pool boundaries or regions with different oxidation states would require spatially resolved or multipoint emissivity measurements.

Figure 16 shows that the retrieved effective emissivity varies smoothly with wavelength over approximately 500–760 nm and also changes with irradiation time. This observation supports the need to account for wavelength dependence rather than applying a single constant emissivity. The curves should, however, be interpreted as representative effective emissivity functions for the sampled region rather than as the true local emissivity of every point in the laser spot.

The temporal emissivity change indicates that the radiative properties of the surface evolved during irradiation. Oxide formation is one physically plausible contributor, but the present dataset does not contain post-irradiation SEM, EDS, XRD, oxide-thickness, or time-resolved reflectance measurements that would identify oxidation as the unique or dominant mechanism. Melting-induced changes in roughness and morphology, local composition changes, and phase evolution may also contribute. The present data therefore support the observation of an emissivity change, while the relative contributions of these mechanisms remain unresolved.

**Figure 17:**
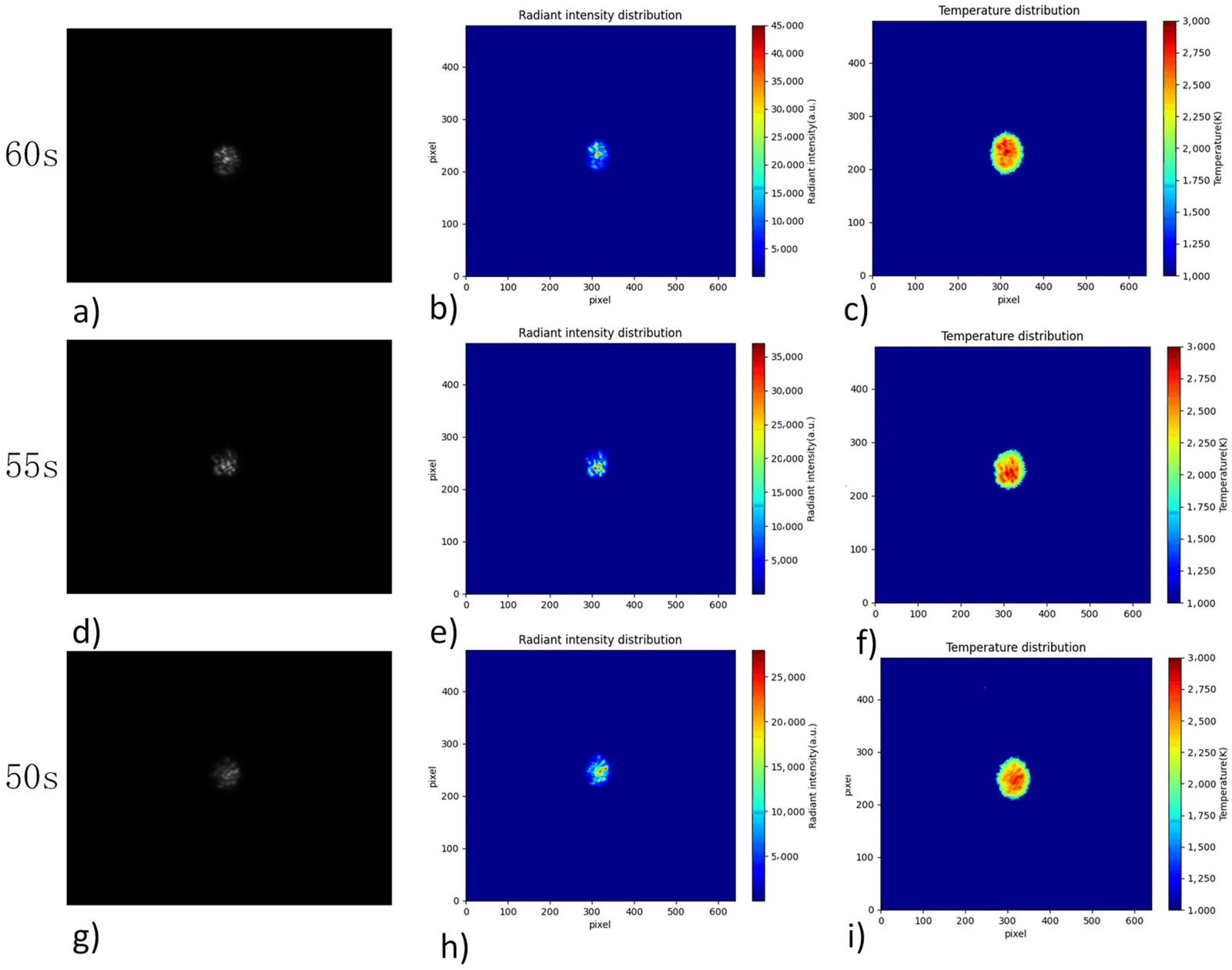
Transient temperature-field inversion: (**a**) gray-level image of the laser-irradiated stainless steel at 60 s; (**b**) radiance distribution at 60 s; (**c**) temperature distribution at 60 s; (**d**) gray-level image at 55 s; (**e**) radiance distribution at 55 s; (**f**) temperature distribution at 55 s; (**g**) gray-level image at 50 s; (**h**) radiance distribution at 50 s; (**i**) temperature distribution at 50 s.

The camera exposure time was set to 0.08 ms to reduce the risk of overexposure and pixel saturation in the high-temperature bright region. The radiometric calibration model was first used to convert each valid pixel gray level to equivalent radiance. The representative emissivity function retrieved from the corresponding spectral measurement (Figure 16) was then introduced into the pixel radiometric equation, and temperature was solved pixel by pixel for 50, 55, and 60 s, as shown in Figure 17. This procedure transfers a representative emissivity function; it does not constitute a spatially resolved emissivity measurement at every pixel.

#### 3.4.2. Spatial Characteristics of the Two-Dimensional Temperature Field

As shown in Figure 17, the reconstructed high-temperature region is concentrated near the laser-beam center and decreases toward the periphery, producing a high-temperature core surrounded by lower-temperature material. The boundary is asymmetric and fluctuating rather than perfectly circular, consistent with the combined effects of beam nonuniformity, surface-state variation, melt-pool motion, and measurement noise. These spatial variations also reinforce the limitation of assuming a single emissivity function for the entire transient field.

From 50 to 60 s, the gray-level images, radiance maps, spectrally retrieved effective temperatures, and reconstructed central high-temperature region show the same temporal ordering. This consistency demonstrates the internal physical coherence of the processing chain comprising spectral inversion, radiometric calibration, representative-emissivity transfer, and pixelwise temperature solution. It should not be interpreted as independent validation of absolute pixel temperature because the same measurement chain supplies the spectral and imaging inputs.

The resulting temperature matrices can also be used to extract quantitative indicators, including peak temperature, regional mean temperature, pixel area above the 1679 K melting point, equivalent high-temperature radius, temperature-centroid position, and radial temperature gradient. These metrics extend qualitative image comparison to quantitative comparisons among different laser powers, interaction times, and material conditions.

#### 3.4.3. Result Reliability, Limitations, and Sources of Error

The synchronized spectral and imaging channels provide complementary point-to-field information. The observed relative spectrum, calibrated spectral radiance, retrieved effective emissivity, spectral-field-of-view temperature, and reconstructed high-temperature region exhibit consistent temporal trends, providing an internal consistency check on the processing chain. The result can nevertheless be affected by system spectral-response calibration, camera nonlinearity, residual dark/background subtraction, saturation, emissivity-model mismatch, temperature-dependent emissivity, non-Lambertian radiation from molten or specular regions, and incomplete overlap between the spectrometer sampling region and the image region of interest. The present data do not allow these contributions to be separated quantitatively.

The transient dataset contains only one irradiation sequence and does not include independent repeated trials, error bars, or a simultaneously measured traceable reference temperature. The reported temperatures should therefore be regarded as estimates produced by the present spectral–image framework; the observed temporal trend and reconstructed spatial evolution are informative, but a traceable absolute-accuracy claim cannot be made from this dataset. Future validation should include at least three independent irradiation repeats under the same conditions, an independently calibrated radiation thermometer or appropriate cross-check, complete dark/background and saturation records, propagation of calibration uncertainty to each pixel, and quantitative characterization of spectral–image registration. Multipoint or region-specific spectral measurements should be added for spatially heterogeneous surfaces. Post-irradiation SEM/EDS/XRD and, where possible, time-resolved reflectance or emissivity measurements should be used to distinguish oxidation, melting, morphology, and phase effects on the evolving radiative properties.

## 4. Conclusions and Outlook

This study developed an integrated spectroscopy–imaging thermometry framework whose central methodological contribution is an integration-time-dependent radiometric calibration model coupled to a representative spectral-emissivity transfer procedure. A second-order emissivity function is retrieved jointly with temperature from calibrated multiwavelength radiance, with the genetic algorithm used as the global optimizer, and the resulting function is then introduced into the imaging equation for pixelwise temperature inversion. The method is explicitly restricted by detector linearity, emissivity-model validity, Lambertian approximation, and spatial representativeness of the spectral sampling region.

In the steady-state tungsten–halogen-lamp experiment, the joint-inversion temperatures and Wien-based spectral-shape estimates exhibited similar values and trends, with a maximum relative deviation of 1.33% between the two estimates. Because both are derived from the same measured spectra, this result is an internal consistency assessment rather than an independent measurement of absolute thermodynamic-temperature accuracy. The nominal lamp color temperatures are likewise treated as operating-condition labels rather than thermodynamic ground truth. In the laser-heated 316L experiment, the spectral-field-of-view effective temperature increased from 2311.92 to 2398.54 K between 50 and 60 s, while the reconstructed images showed a strengthening central high-temperature region. The temperatures above the melting point are consistent with the possible presence of a molten or melt-pool core together with cooler surrounding material, not with a spatially uniform molten field.

The second-order polynomial emissivity model is supported by the AIC-based complexity argument and by the observed reduction in unnormalized steady-state fitting residuals from first to second order. These results support the model for the smooth 500–760 nm spectra examined here, but they do not constitute a complete material-specific order-sensitivity validation for tungsten and 316L. A future reanalysis should report RSS, normalized residual metrics, AIC/BIC, retrieved temperature, and run-to-run optimizer dispersion for first-, second-, and third-order models under identical settings.

The spectral approach reduces reliance on gray-body and fixed emissivity-ratio assumptions by introducing a measured, wavelength-dependent representative emissivity function into the image inversion. It therefore has the potential to reduce systematic bias when constant-emissivity assumptions are inadequate, but it does not eliminate all emissivity-related error: spatial nonuniformity, temperature dependence, phase changes, and directional radiation can still produce bias. The implementation uses one spectrometer and one monochrome imaging camera and is therefore a compact architecture; no universal cost percentage or condition-independent accuracy advantage over commercial multispectral or two-color systems is claimed.

An uncertainty-propagation framework was established to identify the main paths by which spectral inversion, emissivity transfer, image-channel noise, radiometric calibration, background subtraction, nonlinearity, saturation, and spatial registration affect the reconstructed temperature. The present transient dataset does not contain all independent measurements needed for a traceable experiment-specific uncertainty budget, and therefore no combined uncertainty such as ±7 K or a universal 0.3–0.4% accuracy is assigned to the transient results. Quantitative uncertainty should instead be evaluated from repeated measurements and independently characterized calibration and noise terms at the actual operating points.

Within the experimentally established detector linear range, and for targets whose emissivity is spectrally smooth and sufficiently uniform over the transfer region, the proposed framework provides a practical route from representative spectral emissivity to two-dimensional temperature reconstruction. Potential applications include laser material processing, combustion and deflagration diagnostics, metallurgical thermal monitoring, and aerospace thermal-protection testing. Future work should prioritize withheld-integration-time validation, repeated transient experiments, independent traceable radiation thermometry, quantitative spectral–image registration, multipoint emissivity measurements, and SEM/EDS/XRD or complementary surface characterization to resolve the physical origin of time-dependent emissivity changes.

## Figures and Tables

**Table 1:** Accuracy of GA inversion using synthetic data (SiC; 100 Monte Carlo trials at each noise level). The GA stops when either the maximum generation number is reached or the relative change in the best fitness remains below 10^−5^ for 50 consecutive generations. The reported convergence rate is the proportion of independent runs satisfying the fitness-stability criterion and the prescribed numerical tolerance.

Noise Level *σ_n_*	*δ_T_* (Mean)	*δ_T_* (Maximum)	RMSE*_ε_* (Mean)	GA Convergence Rate
0.1%	0.02%	0.05%	$4.2\times 10^{-4}$	100%
0.2%	0.04%	0.11%	$7.8\times 10^{-4}$	100%
0.5%	0.09%	0.28%	$1.9\times 10^{-3}$	100%
1%	0.16%	0.51%	$3.6\times 10^{-3}$	99.9%
2%	0.29%	0.94%	$7.1\times 10^{-3}$	99.6%
5%	0.63%	2.1%	$1.8\times 10^{-2}$	97.2%
10%	1.2%	4.3%	$3.9\times 10^{-2}$	91.4%
20%	2.8%	9.7%	$8.5\times 10^{-2}$	78.6%

**Table 2:** Comparison of the proposed method with commonly used radiation-thermometry methods.

Feature	Proposed Method (Spectrometer + CCD)	Two-Color Pyrometry	Color-Ratio Pyrometry
Hardware configuration	Spectrometer + monochrome CCD	Dual-wavelength detector/camera	Two narrowband filters + CCD
Emissivity treatment	Representative wavelength-dependent emissivity retrieved spectrally and transferred to pixelwise temperature inversion	Gray-body assumption	Emissivity-ratio assumption
Spatial resolution	CCD-pixel level	Depends on the detector array	CCD-pixel level
Applicable temperature range	1500–3000 K (design range of this study)	Depends on the wavelength pair	Depends on the wavelength pair
Primary advantage	Reduces reliance on gray-body or fixed emissivity-ratio assumptions through continuous spectral constraints	Simple configuration and rapid response	Does not require the absolute emissivity
Primary limitation	Requires representative spectral sampling; accuracy is limited by spatial emissivity nonuniformity, calibration, and model validity	Large error when the gray-body assumption is invalid	Residual emissivity error for nongray targets
Accuracy/validation	Condition-dependent; the present experiments do not establish traceable absolute accuracy	Condition-dependent on wavelength pair, calibration, and emissivity behavior	Condition-dependent on channel response, calibration, and emissivity ratio

**Table 3:** Principal hardware specifications of the spectral–image fusion thermometry system.

Hardware Component	Model/Manufacturer	Principal Specifications and Experimental Settings	Experimental Function
Industrial area-array camera	MV-CA003-21UM/HIKROBOT (Hangzhou, Zhejiang, China)	onsemi PYTHON300 monochrome global-shutter CMOS (onsemi, Scottsdale, AZ, USA); 640 × 480 pixels; 4.8 μm × 4.8 μm pixel pitch; 1/4-inch format; maximum frame rate, 814.5 fps; dynamic range, 59 dB; SNR, 39.9 dB; gain, 0–15 dB; exposure time, 40 μs–10 s; USB 3.0; C-mount. Experimental exposure times: 0.04 ms for steady-state measurements and 0.08 ms for transient measurements.	Acquisition of two-dimensional gray-level images for pixelwise temperature inversion using the transferred representative emissivity function
Imaging lens and filter assembly	100 mm telephoto lens/ZLKC (Guangzhou, China) 1000 nm short-pass filter/ZLKC (Guangzhou, China)	f = 100 mm; C-mount; cutoff wavelength = 1000 nm.	Long-distance imaging; >1000 nm suppression; saturation protection
Fiber-optic spectrometer	USB2000+/Ocean Optics (Dunedin, FL, USA)	Sony ILX511B 2048-pixel linear CCD (Sony Semiconductor Solutions Corporation, Atsugi, Kanagawa, Japan); configured range, 200–1100 nm; optical resolution, approximately 1.5 nm (FWHM); 16-bit A/D conversion; integration time, 1 ms–65 s; experimental integration time, 1 ms; corrected linearity > 99.8%.	Acquisition of continuous spectra for joint retrieval of temperature and spectral emissivity after radiometric calibration
Fiber collimator	FC-COL1825/Shenzhen Xinrui Optical Technology Co., Ltd. (Shenzhen, Guangdong, China)	Clear aperture, 18 mm; focal length, 25 mm; mounted with the imaging camera on the same precision adjustment bracket with parallel optical axes.	Collection and coupling of target radiation into the fiber; the reverse-illumination alignment procedure is used to locate the spectral sampling region within the camera field of view
SMA905 energy-transmission fiber	Xinruiguang (Shenzhen, China)	Core diameter, 400 μm; operating range, 400–1100 nm; SMA905 connectors at both ends.	Transmission of radiation collected by the collimator to the spectrometer
Synchronous-trigger module	Custom ESP32 module	Rising-edge trigger; GPIO push-pull output; microsecond-level synchronization accuracy; unified control of camera and spectrometer acquisition timing.	Improvement of temporal correspondence between spectral and image data to support transient temperature-field fusion
Steady-state radiation source	SL3186 standard color-temperature tungsten lamp/YouLai Technology (Hangzhou, China)	Four equivalent color temperatures: 2200, 2400, 2600, and 2800 K.	Generation of a steady-state high-temperature radiation field for algorithm validation and comparison of thermometry results
Pulsed fiber laser	YDFLP-120-LM1-L1/JPT (Shenzhen, China)	MOPA pulsed laser; center wavelength, 1064 nm; average output power > 120 W; maximum single-pulse energy, 1.5 mJ; pulse width, 10–350 ns; repetition rate, 80–1000 kHz over the full-power range; M2 < 1.8; output-beam diameter, 6 ± 0.5 mm; adjustable power, 0–100%; air-cooled; 24 VDC.	Irradiation of 316L stainless steel to generate a transient laser-heating temperature field
Host computer and acquisition software	Lenovo computer and supporting control software	USB 3.0 interface; camera-parameter configuration, spectrometer control, synchronous triggering, raw-data storage, radiometric calibration, and temperature inversion.	System control, data management, and spectral–image fusion thermometry computation

**Table 4:** Comparison of temperature-measurement results.

Thermometry Method	Group 1	Group 2	Group 3	Group 4
Nominal color temperature/K	2200	2400	2600	2800
Emissivity-polynomial method/K	2231.54	2526.79	2696.78	2849.69
Wien displacement method/K	2260	2544	2678	2888

**Table 5:** Principal thermophysical properties of 316L stainless steel.

Material	316L Stainless Steel
Density	8000 kg·m^−3^
Melting point	1679 K
Specific heat capacity	500 J·kg^−1^·K^−1^
Thermal conductivity	16.2 W·m^−1^·K^−1^
Coefficient of thermal expansion	16.0 × 10^−5^ K^−1^

**Table 6:** Retrieved temperatures and temperature-change characteristics at different times.

Time/s	Retrieved Temperature/K	Temperature Rise over Adjacent Interval/K	Mean Heating Rate/(K·s^−1^)	Increase Relative to 50 s/%	Temperature Above the Melting Point/K
50	2311.92	—	—	0	632.92
55	2336.05	24.13	4.83	1.04	657.05
60	2398.54	62.49	12.50	3.75	719.54

## Data Availability

Data underlying the results presented in this paper are not publicly available at this time but may be obtained from the authors upon reasonable request.
